# Immature Seed Endosperm and Embryo Proteomics of the Lotus (*Nelumbo Nucifera* Gaertn.) by One-Dimensional Gel-Based Tandem Mass Spectrometry and a Comparison with the Mature Endosperm Proteome

**DOI:** 10.3390/proteomes3030184

**Published:** 2015-08-14

**Authors:** Carlo F. Moro, Yoichiro Fukao, Junko Shibato, Randeep Rakwal, Ganesh Kumar Agrawal, Seiji Shioda, Yoshiaki Kouzuma, Masami Yonekura

**Affiliations:** 1 Laboratory of Molecular Food Functionality, College of Agriculture, Ibaraki University, Ami, Ibaraki 300-0393, Japan; E-Mails: carlofm@gmail.com (C.F.M.); kouzuma@mx.ibaraki.ac.jp (Y.K.); yonekura@mx.ibaraki.ac.jp (M.Y.); 2 Plant Global Educational Project, Nara Institute of Science and Technology, Ikoma, Nara 630-0192, Japan; E-Mail: y-fukao@fc.ritsumei.ac.jp; 3 Department of Bioinformatics, Ritsumeikan University, Kusatsu, Shiga 525-8577, Japan; 4 Global Research Center for Innovative Life Sciences, Hoshi University School of Pharmacy and Pharmaceutical Sciences, 2-4-41 Ebara, Shinagawa, Tokyo 142-8501, Japan; E-Mails: rjunko@nifty.com (J.S.); shioda@hoshi.ac.jp (S.S.); 5 Faculty of Health and Sport Sciences & Tsukuba International Academy for Sport Studies (TIAS), University of Tsukuba, 1-1-1 Tennodai, Tsukuba, Ibaraki 305-8574, Japan; 6 Research Laboratory for Biotechnology and Biochemistry (RLABB), GPO 13265, Kathmandu 44600, Nepal; 7 GRADE (Global Research Arch for Developing Education) Academy Pvt., Ltd., Adarsh Nagar-13, Birgunj 44300, Nepal

**Keywords:** 1-DGE, LC-MS/MS, lotus, seed, proteome analysis, plant proteomics

## Abstract

Lotus (*Nelumbo nucifera* Gaertn.) seed proteome has been the focus of our studies, and we have recently established the first proteome dataset for its mature seed endosperm. The current study unravels the immature endosperm, as well as the embryo proteome, to provide a comprehensive dataset of the lotus seed proteins and a comparison between the mature and immature endosperm tissues across the seed’s development. One-dimensional gel electrophoresis (SDS-PAGE) linked with tandem mass spectrometry provided a protein inventory of the immature endosperm (122 non-redundant proteins) and embryo (141 non-redundant proteins) tissues. Comparing with the previous mature endosperm dataset (66 non-redundant proteins), a total of 206 non-redundant proteins were identified across all three tissues of the lotus seed. Results revealed some significant differences in proteome composition between the three lotus seed tissues, most notably between the mature endosperm and its immature developmental stage shifting the proteins from nutrient production to nutrient storage.

## 1. Introduction

*Nelumbo nucifera* (Gaertn.) is an aquatic perennial belonging to the family of Nelumbonaceae, whose most used common name is the lotus. The lotus typically grows in shallow ponds, with its rhizomes under the mud and its large leaves rising on stalks 1–2 m above the water surface. Flowers are white to rosy, sweet-scented, solitary, hermaphrodite and 10–25 cm in diameter, while its fruits are ovoid having nut like achenes. Seeds are black, hard and ovoid [1]. In its immature form, the lotus seed is initially of a yellowish color (early stages) and becomes green as it grows and matures. In its late immature stages, the seed is a 1.2–1.5 cm long ovoid covered in a soft green husk containing a moist and soft endosperm and the developing embryo. When the seed reaches maturity, the husk turns dark brown and hardens, and both the endosperm and embryo become considerably dry. The lotus embryo, or germ, is a small, stalk-like tissue at the core of the lotus seed. The embryo is green and yellow in color. In the mature seed, the embryo tissue is dry, and while inside an intact seed, it can remain viable for germination for more than a thousand years, making it the most durable seed known [2,3,4,5]. The immature seed, which is composed largely of the endosperm, has a water content of 77.5%, as opposed to the 13.1% water content of the mature seed. The immature seed also has lower protein and carbohydrate content, 5.9% and14.9%, respectively, compared to 19.1% and 62.6% for the mature seed [6].

**Figure 1:**
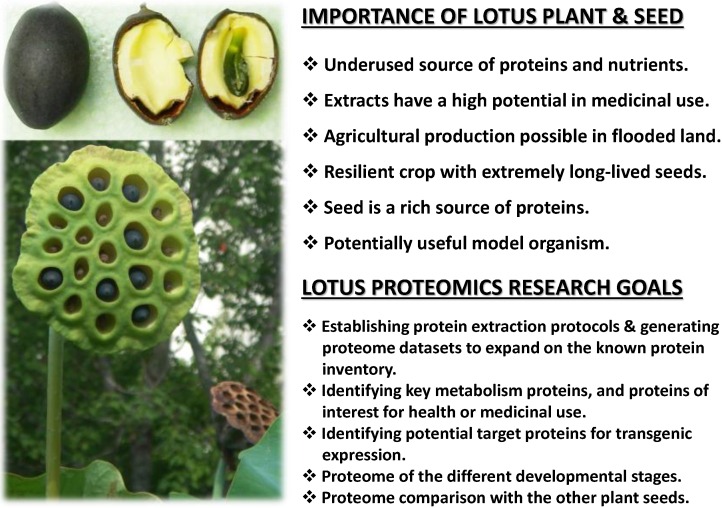
Overview of the significance and goals of the proteomic research of the lotus. The fruit (seedpod) with seeds from a lotus plant growing in Ibaraki University pond, and the open seed with endosperm and embryo is shown.

The lotus seeds and rhizome are extensively consumed as food in China and Japan and regarded as a health food [7,8,9], and the plant is also utilized as a source of traditional medicine in India and China [1,10]. Furthermore, extracts from the lotus leaves, rhizomes, and seeds have been shown possess multiple health benefits and a diverse amount of secondary metabolites (more details are given in our review [11] and references therein). The genome of the lotus has only recently been sequenced [12], and a few targeted genome and transcriptome-level works have led to the identification of some functional proteins, as well as their successful cloning and transgenic expression [13,14,15,16]. Considering its documented health benefits and several desirable characteristics for nutritional, agricultural and scientific uses, such as its protein content, ability to be cultivated in flooded areas, growth and germination vigor, and extreme seed durability, the lotus plant would consist of an excellent candidate as a crop, source of recombinant genes, or even as potential model organism. However, despite these characteristics, proteome analysis of the plant is still at the initial stages of research. Figure 1 depicts the lotus fruit and seed, its importance and proteomic study goals. 

Aiming to develop a proteome catalogue of the lotus plant—starting with its seed, the nutrient rich food source—the first study by our research group has unraveled the mature endosperm proteome of the lotus seed, which included the establishment of protocols for protein extraction and analyses by one-dimensional gel electrophoresis (1-DGE) and by two-dimensional gel electrophoresis (2-DGE) in conjunction with mass spectrometry [17]. In the present work, we advance our study of the lotus seed by further analyzing the endosperm of the lotus seed in its immature stage and the embryo, the other prominent component of the mature seed, by utilizing 1-DGE linked with tandem mass spectrometry proteomic approach. The resulting proteome from each tissue (immature endosperm and embryo) is compared with the mature endosperm proteins in hope to bring to light any notable differences in protein content between the different tissue locations and developmental stages.

## 2. Experimental Section

### 2.1. Plant Material and Tissues (Immature Endosperm and Embryo of Lotus Seed) Preparation

Lotus seeds, both mature and immature, were obtained from a small cultivation pond in the Ibaraki University’s College of Agriculture campus in Ami town, Ibaraki, Japan [17]. The immature seed endosperm was collected from seeds extracted from the lotus seedpod in their post-pollination late immature stage. At the point of collection, the seeds were approximately 1.3 cm long, and the external husk was still green and soft. The seeds were washed and stored whole at −80 °C until tissue extraction. The seeds were cut open and the soft and white core was removed whole and then cut across its length. The translucent sheet around the core, any discernible embryo tissue, as well as the central portion of the core immediately around the embryo was removed. The remaining soft endosperm fragments were ground under liquid nitrogen and the resulting powder was stored in sterile BD Falcon tubes at −80 °C until extraction of protein. For embryo tissue sample preparation, the mature seeds (stored at room temperature) were cracked open in a clean environment and the endosperm and embryo portions were cleanly separated and stored in sterile BD Falcon tubes at −80 °C. The embryo fragments were ground into a fine powder in liquid nitrogen, with a pre-chilled mortar and pestle. Resulting powder was stored in sterile 2.0 mL microfuge tubes at −80 °C until further analysis.

### 2.2. Extraction of the Lotus Seed Immature Endosperm and Embryo Proteins

Proteins were extracted from the powdered samples using the Tris-buffered saline (TBS) extraction method described in a previous study [17]. Briefly, a 3:1 mixture of TBS-20 buffer [10 mM Tris-HCl, 150 mM NaCl, pH 7.4, 0.1% (*v*/*v*) Tween-20, plus one tablet of EDTA-free proteinase inhibitor (cOmplete Mini, Roche) per 50 mL] and SDS (sodium dodecyl sulfate) reducing buffer [62 mM Tris (pH 6.8), 10% (*v*/*v*) glycerol, 2.5% (*w*/*v*) SDS, 5% (*v*/*v*) 2-mercaptoethanol] was used to extract the powdered samples at 2 mL/100 mg. The sample/buffer mixtures were also subjected to several 30 s ultrasonic bath cycles and at 95 °C heating for 5 min to help extraction. The extract was separated by centrifugation, and its proteins precipitated and purified using the ProteoExtract kit (Calbiochem). The dry protein pellets obtained were either resolubilized in LB-TT (7 M urea, 2 M thiourea, 4% (*w*/*v*) CHAPS, 18 mM Tris-HCl (pH 8.0), 14 mM Trizma base, 0.2% (*v*/*v*) Triton X-100 and 50 mM dithiothreitol) for immediate use or stored at −80 °C. Prior to use, protein content of the resolubilized extracts was measured by Bradford assay [18].

### 2.3. Extraction of the Lotus Seed Immature Endosperm and Embryo Proteins

**Figure 2:**
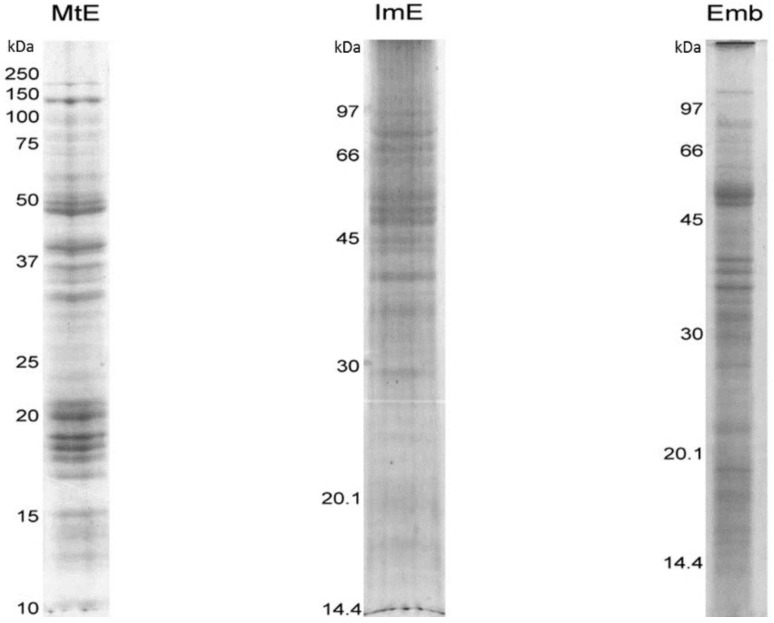
1D SDS-PAGE of protein extracts from lotus seed mature endosperm (MtE), immature endosperm (ImE), and embryo (Emb). SDS-PAGE, 12.5%; Coomassie brilliant blue stained. Molecular weight markers are shown on left-hand side of each gel image.

Protein samples from both tissues were subjected to 1-DGE (SDS-PAGE, 12.5%), both for visualization of protein profiles (Figure 2) using Coomassie Brilliant Blue [19] staining, and prior to analysis by 1DGE-MS.

The 1DGE-MS analyses followed the same methodology as with the previous lotus seed analyses [17]. The extracts were initially separated using SDS-PAGE. The resulting vertical protein lanes were sliced into eight pieces of equal length (regardless of apparent protein concentration) giving fraction 1: <120 kDa, fraction 2: 120–60 kDa, fraction 3: 60–40 kDa, fraction 4: 40–30 kDa, fraction 5: 30–22 kDa, fraction 6: 22–17 kDa, fraction 7: 17–14 kDa, and fraction 8: 14–10 kDa. Each fraction was digested with 1 µg of trypsin at 37 °C for 16 h [17,18,19,20]. Digested peptides were recovered twice with 20 µL of 5% (*v*/*v*) formic acid in 50% (*v*/*v*) acetonitrile. Extracted peptides were combined and then evaporated in a vacuum concentrator until liquid was dry. Dried peptides were dissolved into 20 µL of 5% acetonitrile/0.1% formic acid and then filtrated by the Ultrafree-MC Centrifugal Filters (Millipore, PVDF 0.45 µm, Darmstadt, Germany). Liquid chromatography–tandem mass spectrometry (MS/MS) analysis was performed using the LTQ-Orbitrap XL-HTC-PAL system (Thermo, Waltham, MA, USA). Trypsin digests were loaded on the column (100 µm internal diameter, 15 cm length, l-Column, CERI) using the Paradigm MS4 HPLC pump (Michrom BioResources, Auburn, AL, USA) and HTC-PAL Autosampler (CTC Analytics, Zwingen, Switzerland), and were eluted by a gradient of 5%–45% (*v*/*v*) acetonitrile in 0.1% (*v*/*v*) formic acid for 26 min. The eluted peptides were introduced directly into an LTQ-Orbitrap with a flow rate of 500 nL/min, and a spray voltage of 2.0 kV. The range of MS scan was *m*/*z* 450–1500. The top three peaks were subjected to MS/MS analysis. MS/MS spectra were analyzed by Mascot server (version 2.4.1, Matrix Science, Boston, MA, USA) in house (http://www.matrixscience.com/) and compared against proteins registered in the SwissProt (SwissProt_2012_03) database (total sequences: 428650; sequences after taxonomy filter (Viridiplantae): 27008; date: 26 July 2013). The Mascot search parameters were set as follows: threshold of the ion score cutoff, 0.05, peptide tolerance, 10 ppm, MS/MS tolerance, 0.5 Da, and peptide charge, 2+ or 3+. The search was also set to allow one missed cleavage by trypsin, a carboxymethylation modification of Cys residues, and a variable oxidation modification of Met residues. Gene ontology analysis on the data was performed using the Uniprot (www.uniprot.org) and the EMBL-EBI (www.ebi.ac.uk) databases.

## 3. Results and Discussion

### 3.1. Protein Content of the Immature Endosperm and Embryo Tissues

Protein extracts from the lotus immature seed endosperm presented very low protein yield (*ca.* 1.5% in the TBS method), requiring larger amounts of tissue to be extracted in order to obtain a suitable amount of protein. The reason for low protein yield lies in the high water content of the immature seed compared to its mature form. The lotus seed embryo showed a similar total protein yield to the endosperm extract [17] when extracted by the TBS/clean-up method (*ca.* 9%, compared to *ca.* 11% for the mature endosperm).

A comparison of the of the 1-D band profile on the SDS-PAGE of the embryo extract with the endosperm one showed many similarities, but also some noticeable differences, such as an absence of strongly stained bands at *ca.* 20 kDa and 40 kDa, and more numerous bands at low-molecular weights, under 30 and 20 kDa (see above, Figure 2). In the case of the immature endosperm, the 1-D profile is more similar to the mature endosperm than the embryo, but still was found to be different from both tissues profiles. Compared with the mature endosperm extract, the immature endosperm extract most notably does not present a high amount of protein bands around the 20 kDa range. The cluster of bands around 50 kDa is similar to that in both the endosperm and embryo, and the immature endosperms profile of bands in the 60–90 kDa range seems more similar to the mature endosperm than the embryo.

### 3.2. Lotus Immature Endosperm Proteins Identified by 1-DGE and MS/MS Analyses

The 1-DGE separation (SDS-PAGE) of proteins in an extract, followed by MS/MS analysis is part of the so-called “bottom-up” approach to proteomics, a methodology in which proteins are proteolytically digested into peptides prior to mass spectrometric analysis, and the ensuing peptide masses and sequences are used to identify corresponding proteins. This simple approach is a useful method for performing large-scale analyses of complex samples [21]. For the sample consisting of a purified extract of lotus immature endosperm proteins, after separation by SDS-PAGE, the sample was divided into eight fractions, analyzed by LC-MS/MS, and matched against a green plant database, as detailed in the Experimental Section. Results revealed more than 500 protein matches with at least two confirmed peptide fragment matches were identified amongst all fractions, and from these 333 unique protein matches were identified. Different database matches that were likely to refer to the same protein in the sample, such as two or more matches for the same protein but from different database organisms, were grouped together based on taxonomical proximity and similarity of identified peptide sequences. Finally, 122 non-redundant (nr) protein matches were listed, along with the number of repeated matches found for each one (Table 1), with the protein match listed being the one with the highest score amongst its group of similar proteins.

### 3.3. Lotus Embryo Proteins Identified by 1-DGE and MS/MS Analyses

The 1-DGE-MS analysis of the lotus embryo protein extract was performed following the same methodology, green plant database, and same parameters as for the immature endosperm extract. For the sample consisting of a purified extract of lotus embryo proteins, after separation by SDS-PAGE, the sample was divided into eight fractions, analyzed by LC-MS/MS, and matched against a green plant database, as above. From the initial results, 500+ protein matches with at least two confirmed peptide fragment matches were identified. After removing duplicate results from different gel fractions, there were 373 unique protein matches remaining. After grouping results likely to be the same protein in the sample, based on protein taxonomy and similarity of identified peptide sequences, 141 nr protein matches were listed (Table 2).

**Table 1:** List of top-scored non-redundant (nr) protein matches of the lotus immature endosperm 1-D shotgun mass spectroscopy results, as matched to Green Plant proteome database (SwissProt 57.0, http://www.uniprot.org/statistics/UniProtKB%2015).

Fractions ^1^	Protein Accession	Protein Description	Similar ^2^	Score ^3^	Cover (%)	PEPTIDE Sequences	Sig. Peptide Number	Func. Cat. ^4^
6,5,8,(7,4,3,1)	ENO1_HEVBR	Enolase 1 OS = *Hevea brasiliensis*	11	1471	43.4	TAIAK, YNQLLR, LTSEIGEK, ACNALLLK, DGGSDYLGK, AGWGVMASHR, EKACNALLLK, MGAEVYHHLK, RAGWGVMASHR, LGANAILAVSLAVCK, VQIVGDDLLVTNPK, AAVPSGASTGIYEALELR, LAMQEFMILPVGASSFK, SGETEDTFIADLSVGLATGQIK, YGQDATNVGDEGGFAPNIQENK, KYGQDATNVGDEGGFAPNIQENK, YGQDATNVGDEGGFAPNIQENKEGLELLK	17	II
7,8,6,5,1,4,2	G3PC_ANTMA	Glyceraldehyde-3-phosphate dehydrogenase, cytosolic OS = *Antirrhinum majus*	23	1242	43.6	AAAHLK, KATYEQIK, AAIKEESEGK, AGIALNDNFVK, DAPMFVVGVNEK, AASFNIIPSSTGAAK, VPTVDVSVVDLTVR, DAPMFVVGVNEKEYK, VPTVDVSVVDLTVRLEK, FGIVEGLMTTVHSITATQK, GILGYTEDDVVSTDFVGDSR, LTGMSFRVPTVDVSVVDLTVR, LKGILGYTEDDVVSTDFVGDSR, VINDRFGIVEGLMTTVHSITATQK	14	II
4,8,6,7,5	HSP7D_ARATH	Heat shock 70 kDa protein 4 OS = *Arabidopsis thaliana*	10	625	23.8	IEEVD, LSKEEIEK, ITITNDKGR, DAGVISGLNVMR, NALENYAYNMR, MVNHFVQEFKR, TTPSYVAFTDSER, IINEPTAAAIAYGLDK, ATAGDTHLGGEDFDNR, NAVVTVPAYFNDSQR, IINEPTAAAIAYGLDKK, EQIFSTYSDNQPGVLIQVYEGER	12	IX
4	HSP7E_SPIOL	Chloroplast envelope membrane 70 kDa heat shock-related protein OS = *Spinacia oleracea*	1	580	21.7	LSKEEIEK, DAGVISGLNVMR, EIAEAYLGSTVK, NALENYAYNMR, TTPSYVAFTDSER, IINEPTAAAIAYGLDK, ATAGDTHLGGEDFDNR, NAVVTVPAYFNDSQR, IINEPTAAAIAYGLDKK, EQVFSTYSDNQPGVLIQVYEGER	10	IX
4	BIP4_TOBAC	Luminal-binding protein 4 OS = *Nicotiana tabacum*	5	542	21.6	VQQLLK, NTVIPTKK, IMEYFIK, LSQEEIER, ITITNDKGR, DYFDGKEPNK, FEELNNDLFR, EAEEFAEEDKK, IVNKDGKPYIQVK, ARFEELNNDLFR, NGHVEIIANDQGNR, IINEPTAAAIAYGLDK, IINEPTAAAIAYGLDKK, IKDAVVTVPAYFNDAQR	14	IX
4,5,6,7,8	METE_ARATH	5-methyltetrahydropteroyltriglutamate—homocysteine methyltransferase OS = *Arabidopsis thaliana*	4	516	19.2	AAAALK, VVEVNALAK, SWLAFAAQK, AVNEYKEAK, YLFAGVVDGR, SDEKLLSVFR, FALESFWDGK, GNASVPAMEMTK, YGAGIGPGVYDIHSPR, GMLTGPVTILNWSFVR	10	I
6,1,5,(7,8,3,2,4)	EF1A_TOBAC	Elongation factor 1-alpha OS = *Nicotiana tabacum*	8	484	34.7	YDEIVK, GFVASNSK, QTVAVGVIK, EVSSYLKK, LPLQDVYK, ARYDEIVK, IGGIGTVPVGR, STNLDWYK, STTTGHLIYK, EHALLAFTLGVK, GFVASNSKDDPAK, YYCTVIDAPGHR, MIPTKPMVVETFSEYPPLGR, NMITGTSQADCAVLIIDSTTGGFEAGISK	14	V
4	HSP7L_ARATH	Heat shock 70 kDa protein 12 OS = *Arabidopsis thaliana*	1	479	16.5	VQQLLK, NTVIPTKK, IMEYFIK, FDLTGVPPAPR, FEELNNDLFR, EAEEFAEEDKK, ARFEELNNDLFR, NGHVEIIANDQGNR, IINEPTAAAIAYGLDK, IINEPTAAAIAYGLDKK, IKDAVVTVPAYFNDAQR	11	IX
4,8,6	HSP7N_ARATH	Heat shock 70 kDa protein 18 OS = *Arabidopsis thaliana*	1	474	18.5	ITITNDKGR, EIAEAYLGSSIK, MVNHFVQEFKR, TTPSYVAFTDSER, IINEPTAAAIAYGLDK, ATAGDTHLGGEDFDNR, NAVVTVPAYFNDSQR, IINEPTAAAIAYGLDKK	8	IX
7	MDHM_CITLA	Malate dehydrogenase, mitochondrial OS = *Citrullus lanatus*	5	459	18.2	TFYAGK, LFGVTTLDVVR, TQDGGTEVVEAK, DDLFNINAGIVK, KLFGVTTLDVVR, RTQDGGTEVVEAK, VAVLGAAGGIGQPLALLMK, KVAVLGAAGGIGQPLALLMK	8	II
4,5	HSP80_SOLLC	Heat shock cognate protein 80 OS = *Solanum lycopersicum*	1	427	20.9	AVENSPFLEK, LGIHEDSQNR, ADLVNNLGTIAR, KAVENSPFLEK, HFSVEGQLEFK, GIVDSEDLPLNISR, SLTNDWEEHLAVK, SGDEMTSLKDYVTR, KPEEITKEEYAAFYK, MKEGQNDIYYITGESK	10	IX
5	CH62_MAIZE	Chaperonin CPN60-2, mitochondrial OS = *Zea mays*	6	422	23.3	VTDALNATK, GVEELADAVK, IGGASEAEVGEK, SVAAGMNAMDLR, IGGASEAEVGEKK, NVVIEQSFGAPK, AAVEEGIVPGGGVALLYASK, TPVHTIASNAGVEGAVVVGK, QRPLLIVAEDVESEALGTLIINK	9	IX
7,8,6,1	ACT_GOSHI	Actin OS = *Gossypium hirsutum*	19	414	32.6	AGFAGDDAPR, GYSFTTTAER, EITALAPSSMK, DAYVGDEAQSK, AVFPSIVGRPR, DAYVGDEAQSKR, SYELPDGQVITIGAER, VAPEEHPVLLTEAPLNPK, TTGIVLDSGDGVSHTVPIYEGYALPHAILR	9	VII
5,8,7	ENO2_ARATH	Bifunctional enolase 2/transcriptional activator OS = *Arabidopsis thaliana*	1	411	34.5	YNQLLR, DGGSDYLGK, ISGDALKDLYK, LGANAILAVSLAVCK, VNQIGSVTESIEAVK, TYDLNFKEENNNGSQK, SGETEDTFIADLAVGLSTGQIK, YGQDATNVGDEGGFAPNIQENK, YGQDATNVGDEGGFAPNIQENKEGLELLK	9	IV
4,5,6	HSP83_IPONI	Heat shock protein 83 OS = *Ipomoea nil*	1	405	23	VIVTTK, VVVSDR, AILFVPK, DVDGEQLGR, APFDLFDTR, AVENSPFLER, LGIHEDSQNR, LDAQPELFIR, RAPFDLFDTR, ADLVNNLGTIAR, ELISNASDALDK, HFSVEGQLEFK, GVVDSDDLPLNISR, ELISNASDALDKIR, ITLFLKEDQLEYLEER	14	IX
7,6,1,2	ADH1_SOLTU	Alcohol dehydrogenase 1 OS = *Solanum tuberosum*	7	390	23.7	ELELEK, SDIPSVVEK, FGVTEFVNPK, GTFFGNYKPR, THPMNLLNER, KFGVTEFVNPK, YMNKELELEK, TLKGTFFGNYKPR, GSSVAIFGLGAVGLAAAEGAR	9	II
4,5,7,8	ENPL_CATRO	Endoplasmin homolog OS = *Catharanthus roseus*	3	387	10.5	FWNEFGK, ESFKELTK, YGWSSNMER, ELISNASDALDK, IMQSQTLSDASK, GLVDSDTLPLNVSR, ELISNASDALDKIR, VFISDEFDELLPK, RVFISDEFDELLPK	9	IX
7,(8,6)	RBL_MAIZE	Ribulose bisphosphate carboxylase large chain OS = *Zea mays*	52	382	27.5	AMHAVIDR, AQAETGEIK, DTDILAAFR, DDFIEKDR, VALEACVQAR, EITLGFVDLLR, LTYYTPEYETK, MSGGDHIHSGTVVGK, YGRPLLGCTIKPK, GGLDFTKDDENVNSQPFMR	10	I
6	SAHH_MEDSA	Adenosylhomocysteinase OS = *Medicago sativa*	3	369	15.1	ATDVMIAGK, HSLPDGLMR, ITIKPQTDR, TEFGPSQPFK, VAVVCGYGDVGK, SKFDNLYGCR, IVGVSEETTTGVK, IVGVSEETTTGVKR	8	I
4,(5,6)	HSP82_ORYSJ	Heat shock protein 81-2 OS = *Oryza sativa* subsp*. Japonica*	2	368	21.5	VVVSDR, IAELLR, AILFVPK, APFDLFDTR, AVENSPFLEK, RAPFDLFDTR, KAVENSPFLEK, SDLVNNLGTIAR, HFSVEGQLEFK, GIVDSEDLPLNISR, SLTNDWEEHLAVK, HSEFISYPISLWTEK, KPEEITKEEYAAFYK	12	IX
4,6,7	HSP70_DAUCA	Heat shock 70 kDa protein OS = *Daucus carota*	1	335	15.7	IEEVD, NALENYAYNMR, NQVAMNPSNTVFDAK, NQVAMNPSNTVFDAKR, SINPDEAVAYGAAVQAAILSGEGNER, EQIFSTYSDNQPGVLIQVYEGER	6	IX
5	CPNA1_ARATH	Chaperonin 60 subunit alpha 1, chloroplastic OS = *Arabidopsis thaliana*	1	331	16.4	KVTISK, VVNDGVTIAR, NVVLDEFGSPK, VGAATETELEDR, GYISPQFVTNPEK, TNDSAGDGTTTASILAR	6	IX
4,5	HSP82_MAIZE	Heat shock protein 82 OS = *Zea mays*	2	297	14.4	APFDLFDTR, AVENSPFLER, LGIHEDSQNR, RAPFDLFDTR, SDLVNNLGTIAR, ELISNASDALDK, HFSVEGQLEFK, GVVDSDDLPLNISR, ELISNASDALDKIR	9	IX
8,7	ALF_CICAR	Fructose-bisphosphate aldolase, cytoplasmic isozyme OS = *Cicer arietinum*	2	289	14.2	ANSEATLGTYK, GILAADESTGTIGK, GILAADESTGTIGKR, YHDELIANAAYIGTPGK	4	II
4,(3,5)	CD48A_ARATH	Cell division control protein 48 homolog A OS = *Arabidopsis thaliana*	3	285	14	TLLAK, KGDLFLVR, ELVELPLR, LAEDVDLER, LAGESESNLR, GILLYGPPGSGK, IVSQLLTLMDGLK, ELVELPLRHPQLFK, NAPSIIFIDEIDSIAPK	9	III/IV
7,8,6,1	PGKH_TOBAC	Phosphoglycerate kinase, chloroplastic OS = *Nicotiana tabacum*	6	284	15.4	AAVPTIK, AHASTEGVTK, FAVGTEAIAK, VILSSHLGRPK, GVTTIIGGGDSVAAVEK, LASLADLYVNDAFGTAHR, KLASLADLYVNDAFGTAHR	7	II
5,(4)	PGMC_POPTN	Phosphoglucomutase, cytoplasmic OS = *Populus tremula*	1	281	12.2	YLFEDGSR, FFEVPTGWK, LSGTGSEGATIR, SMPTSAALDVVAK, YDYENVDAGAAK, VETTPFGDQKPGTSGLR	6	II
4,(5)	HS903_ARATH	Heat shock protein 90-3 OS = *Arabidopsis thaliana*	3	269	20.2	IAELLR, AILFVPK, AVENSPFLEK, LGIHEDSQNR, ADLVNNLGTIAR, KAVENSPFLEK, HFSVEGQLEFK, GIVDSEDLPLNISR, HSEFISYPISLWIEK	9	IX
5,6	PMG2_ARATH	Probable 2,3-bisphosphoglycerate-independent phosphoglycerate mutase 2 OS = *Arabidopsis thaliana*	1	257	13.6	VHILTDGR, ARDAILSGK, LVDLALASGK, TFACSETVK, MKALEIAEK, GWDAQVLGEAPHK, RGWDAQVLGEAPHK, AVGPIVDGDAVVTFNFR	8	II
5,6	PMG1_ARATH	2,3-bisphosphoglycerate-independent phosphoglycerate mutase 1 OS = *Arabidopsis thaliana*	5	224	10.2	VHILTDGR, ARDAILSGK, LDQLQLLIK, GWDAQVLGEAPHK, RGWDAQVLGEAPHK, AVGPIVDGDAVVTFNFR	6	II
5	SSG1_HORVU	Granule-bound starch synthase 1, chloroplastic/amyloplastic OS = *Hordeum vulgare*	5	214	7.5	FFHCYK, EALQAEVGLPVDR, FSLLCQAALEAPR, VAFCIHNISYQGR	4	I
6	RL4_PRUAR	60S ribosomal protein L4 OS = *Prunus armeniaca*	3	200	20.8	AGQGAFGNMCR, AGHQTSAESWGTGR, YAVVSAIAASAVPSLVLAR, AWYQTMISDSDYTEFDNFTK	4	V
8	H2B_GOSHI	Histone H2B OS = *Gossypium hirsutum*	5	200	49	IYIFK, LVLPGELAK, AMGIMNSFINDIFEK	3	VII
5	RUBA_RICCO	RuBisCO large subunit-binding protein subunit alpha (Fragment) OS = *Ricinus communis*	2	200	16	NVVLDEFGSPK VGAATETELEDR, GYISPQFVTNPEK, LGLLSVTSGANPVSIK	4	I
8	H2B1_MEDTR	Probable histone H2B.1 OS = *Medicago truncatula*	2	199	45.3	IYIFK, LVLPGELAK, AMGIMNSFINDIFEK	3	VII
8	RL182_ARATH	60S ribosomal protein L18-2 OS = *Arabidopsis thaliana*	1	185	13.4	APLGQNTVLLR, AGGECLTFDQLALR	2	V
5	CALR_BERST	Calreticulin OS = *Berberis stolonifera*	3	175	9.1	LAEETWGK, LLSGDVDQK, KLAEETWGK, TLVFQFSVK, LLSGDVDQKK, YVGIELWQVK	6	V
8	1433E_TOBAC	14-3-3-like protein E OS = *Nicotiana tabacum*	5	172	26.8	NVIGAR, NLLSVAYK, DSTLIMQLLR, TVDVEELTVEER, IISSIEQKEESR, SAQDIALAELAPTHPIR	6	VIII
8	H4_ARATH	Histone H4 OS = *Arabidopsis thaliana*	1	160	45.6	TLYGFGG, IFLENVIR, DAVTYTEHAR, ISGLIYEETR, DNIQGITKPAIR	5	VII
6,5,8	KPYC_SOYBN	Pyruvate kinase, cytosolic isozyme OS = *Glycine max*	2	157	9.2	KGSDLVNVR, GDLGMEIPVEK, VENQEGVLNFDEILR	3	II
8	RS6_ASPOF	40S ribosomal protein S6 OS = *Asparagus officinalis*	2	155	11.6	LVTPLTLQR, ISQEVSGDALGEEFK, ISQEVSGDALGEEFKGYVFK	3	V
5	TCPA_ARATH	T-complex protein 1 subunit alpha OS = *Arabidopsis thaliana*	1	153	6.4	YFVEAGAIAVR, VLVELAELQDR, NKIHPTSIISGYR	3	V
1	ADT1_GOSHI	ADP, ATP carrier protein 1, mitochondrial OS = *Gossypium hirsutum*	1	143	5.7	SSLDAFSQILK, LLIQNQDEMIK	2	VI
4	HSP7S_SPIOL	Stromal 70 kDa heat shock-related protein, chloroplastic (Fragment) OS = *Spinacia oleracea*	2	142	7.2	QFAAEEISAQVLR, AVVTVPAYFNDSQR, IINEPTAASLAYGFEK	3	IX
7,8	GCST_PEA	Aminomethyltransferase, mitochondrial OS = *Pisum sativum*	2	140	14.7	LYFGEFR, GGAIDDSVITK, SLLALQGPLAAPVLQHLTK, TGYTGEDGFEISVPSEHGVELAK	4	I
3,2	HSP7O_ARATH	Heat shock 70 kDa protein 14 OS = *Arabidopsis thaliana*	1	139	7.7	ILSHAFDR, AVLDAATIAGLHPLR, AVEKEFEMALQDR, RAVLDAATIAGLHPLR	4	IX
4	HSP7F_ARATH	Heat shock 70 kDa protein 6, chloroplastic OS = *Arabidopsis thaliana*	1	139	7.5	TTPSVVAYTK, QFAAEEISAQVLR, QAVVNPENTFFSVK, LSFKDIDEVILVGGSTR	4	IX
8	RS4_GOSHI	40S ribosomal protein S4 OS = *Gossypium hirsutum*	2	135	20.6	LSIIEEAR, LGNVFTIGK, FDVGNVVMVTGGR, LGGAFAPKPSSGPHK	4	V
3,4	CLPA_BRANA	ATP-dependent Clp protease ATP-binding subunit clpA homolog, chloroplastic (Fragment) OS = *Brassica napus*	1	135	5.8	VIGQDEAVK, TAIAEGLAQR, YRGEFEER, VLELSLEEAR	4	I
4,5,8	EF2_BETVU	Elongation factor 2 OS = *Beta vulgaris*	1	131	5.5	GGGQIIPTAR, EGALAEENMR, RVFYASQLTAKPR, LWGENFFDPATKK	4	V
8	RL12_PRUAR	60S ribosomal protein L12 OS = *Prunus armeniaca*	1	129	22.3	VSVVPSAAALVIK, VTGGEVGAASSLAPK	2	V
6,(7,8)	ATPBM_NICPL	ATP synthase subunit beta, mitochondrial OS = *Nicotiana plumbaginifolia*	4	129	12.5	VLNTGSPITVPVGR, TVLIMELINNVAK, IPSAVGYQPTLATDLGGLQER	3	I
4,(7)	PHSH_SOLTU	Alpha-glucan phosphorylase, H isozyme OS = *Solanum tuberosum*	6	125	5.6	AFATYTNAK, QLLNILGVIYR, HMEIIEEIDKR, TIAYTNHTVLPEALEK	4	II
6	RL3_ORYSJ	60S ribosomal protein L3 OS = *Oryza sativa* subsp. *Japonica*	2	124	6.9	VIAHTQIR, HGSLGFLPR, GKGYEGVVTR	3	V
8	TPIS_MAIZE	Triosephosphate isomerase, cytosolic OS = *Zea mays*	2	124	11.9	FFVGGNWK, VAYALSQGLK, VIACVGETLEQR	3	VII
8	LE194_HORVU	Late embryogenesis abundant protein B19.4 OS = *Hordeum vulgare*	1	121	9.2	GGLSTMNESGGER, KGGLSTMNESGGER	2	IX
1,(2)	AVP_VIGRR	Pyrophosphate-energized vacuolar membrane proton pump OS = *Vigna radiata* var. *Radiata*	2	117	3.3	AADVGADLVGK, YIEAGASEHAR, AADVGADLVGKVER	3	VI
8	RL6_MESCR	60S ribosomal protein L6 OS = *Mesembryanthemum crystallinum*	1	115	10.7	VDISGVNVEK, ASITPGTVLIILAGR	2	V
5	SSG1_ARATH	Probable granule-bound starch synthase 1, chloroplastic/amyloplastic OS = *Arabidopsis thaliana*	1	115	3.9	FFHCYK, YGTVPIVASTGGLVDTVK	2	I
8	RL10_VITRI	60S ribosomal protein L10 OS = *Vitis riparia*	1	114	10.5	VSIGQVLLSVR, ENVSSEALEAAR	2	V
8	RS18_ARATH	40S ribosomal protein S18 OS = *Arabidopsis thaliana*	1	111	21.7	LRDDLER, VLNTNVDGK, IMFALTSIK, IPDWFLNR	4	V
7	AATM_LUPAN	Aspartate aminotransferase P2, mitochondrial (Fragment) OS = *Lupinus angustifolius*	1	111	6.8	IADVIQEK, LNLGVGAYR, VATVQGLSGTGSLR	3	I
8	GBLPA_ORYSJ	Guanine nucleotide-binding protein subunit beta-like protein A OS = *Oryza sativa* subsp. *Japonica*	1	110	9	DGVTLLWDLAEGK, FSPNTFQPTIVSGSWDR	2	VIII
8	H2AX_CICAR	Histone H2AX OS = *Cicer arietinum*	1	108	15.1	AGLQFPVGR, GKGEIGSASQEF	2	VII
4	VATA_GOSHI	V-type proton ATPase catalytic subunit A OS = *Gossypium hirsutum*	2	108	5	LAADTPLLTGQR, LVSQKFEDPAEGEEALVAK	2	VI
8	PARP3_SOYBN	Poly [ADP-ribose] polymerase 3 OS = *Glycine max*	1	107	4.2	VLCSQEIYK, LEPLVANFMK, LFEEITGNEFEPWER	3	III
3,4,(5)	CLPC1_ARATH	Chaperone protein ClpC1, chloroplastic OS = *Arabidopsis thaliana*	5	106	5.4	TAIAEGLAQR, YRGEFEER, VLELSLEEAR	3	IX
8	NDK1_ARATH	Nucleoside diphosphate kinase 1 OS = *Arabidopsis thaliana*	2	103	9.4	NVIHGSDSVESAR, NVIHGSDSVESARK	2	I
8	RL13_TOBAC	60S ribosomal protein L13 OS = *Nicotiana tabacum*	1	98	16.3	SLEGLQTNVQR, KLAPTIGIAVDHR	2	V
8	RS5_CICAR	40S ribosomal protein S5 (Fragment) OS = *Cicer arietinum*	2	95	15.2	GSSNSYAIK, AQCPIVER, VNQAIYLLTTGAR	3	V
5,6	PDC2_ORYSI	Pyruvate decarboxylase isozyme 2 OS = *Oryza sativa* subsp. *Indica*	2	94	4.5	AVKPVLVGGPK, ILHHTIGLPDFSQELR	2	II
8	HSP14_SOYBN	17.5 kDa class I heat shock protein OS = *Glycine max*	4	92	24.7	AIEISG, ADIPGLK, VLQISGER, FRLPENAK	4	IX
6	AMPL1_ARATH	Leucine aminopeptidase 1 OS = *Arabidopsis thaliana*	2	92	4.6	GLTFDSGGYNIK, TIEVNNTDAEGR	2	I/IX
6	ACT5_ARATH	Putative actin-5 OS *= Arabidopsis thaliana*	1	92	15.9	AGFAGDDAPR, IWHHTFYNELR	2	VII
8	RS14_CHLRE	40S ribosomal protein S14 OS = *Chlamydomonas reinhardtii*	1	87	15.7	TPGPGAQSALR, IEDVTPIPTDSTR	2	V
8	RS3A1_VITVI	40S ribosomal protein S3a-1 OS = *Vitis vinifera*	2	86	6.5	TTDNYTLR, LRAEDVQGR	2	V
7	AAT3_ARATH	Aspartate aminotransferase, chloroplastic OS = *Arabidopsis thaliana*	1	83	4.9	LNLGVGAYR, TEEGKPLVLNVVR	2	I
1	COB21_ORYSJ	Coatomer subunit beta-1 OS = *Oryza sativa* subsp. *Japonica*	1	83	4.5	HNEIQTVNIK, DTNTFASASLDR	2	VI
8	GRDH1_ARATH	Glucose and ribitol dehydrogenase homolog 1 OS = *Arabidopsis thaliana*	3	83	8	GAIVAFTR, EGSSIINTTSVNAYK	2	II
8	ANXD1_ARATH	Annexin D1 OS = *Arabidopsis thaliana*	1	80	5	AQINATFNR, SKAQINATFNR	2	IX
7	PDI21_ORYSJ	Protein disulfide isomerase-like 2-1 OS = *Oryza sativa* subsp. *Japonica*	1	80	10.9	KLAPEYEK, YGVSGFPTLK, YGVSGYPTIQWFPK	3	V
8,(6)	ATPAM_NICPL	ATP synthase subunit alpha, mitochondrial OS = *Nicotiana plumbaginifolia*	4	79	9.4	VVSVGDGIAR, TAIAIDTILNQK	2	I
8,(1)	CB2_PHYPA	Chlorophyll a-b binding protein, chloroplastic OS = *Physcomitrella patens* subsp. *Patens*	1	75	3.7	ELEVIHAR, NRELEVIHAR	2	II
8	HSP12_SOYBN	Class I heat shock protein (Fragment) OS = *Glycine max*	1	75	18.9	AIEISG, ILQISGER	2	IX
8	BAS1_ORYSJ	2-Cys peroxiredoxin BAS1, chloroplastic OS = *Oryza sativa* subsp. *Japonica*	1	69	9.6	LSDYIGKK, SGGLGDLKYPLISDVTK	2	IX
8	RLA0_LUPLU	60S acidic ribosomal protein P0 OS = *Lupinus luteus*	1	69	7.5	VGSSEAALLAK, GTVEIITPVELIK	2	V
7	EF1G2_ORYSJ	Elongation factor 1-gamma 2 OS = *Oryza sativa* subsp. *Japonica*	1	68	5	NPLDLLPPSK, SFTSEFPHVER	2	V
1	MDAR_SOLLC	Monodehydroascorbate reductase OS = *Solanum lycopersicum*	1	68	9.7	AYLFPEGAAR, IVGAFLESGSPEENKAIAK	2	IX
7	RSSA_BRANA	40S ribosomal protein SA OS = *Brassica napus*	1	65	10.3	LLILTDPR, VIVAIENPQDIIVQSARPYGQR	2	V
6,8	IF4A1_ARATH	Eukaryotic initiation factor 4A-1 OS = *Arabidopsis thaliana*	1	65	8.3	ELAQQIEK, VLITTDLLAR	2	V
7	HSP11_PEA	18.1 kDa class I heat shock protein OS = *Pisum sativum*	1	64	14.6	SIEISG, VLQISGER	2	IX
8	RS16_FRIAG	40S ribosomal protein S16 OS = *Fritillaria agrestis*	1	64	12.4	ALVAYYQK, AFEPILLLGR	2	V
8	RL51_ARATH	60S ribosomal protein L5-1 OS = *Arabidopsis thaliana*	1	64	7.3	KLTYEER, GALDGGLDIPHSDKR	2	V
4	HSP7M_PHAVU	Heat shock 70 kDa protein, mitochondrial OS = *Phaseolus vulgaris*	1	64	6.1	HLNITLTR, SSGGLSEDEIEK	2	IX
8,7	HSP12_MEDSA	18.2 kDa class I heat shock protein OS = *Medicago sativa*	1	63	22.8	TIDISG, VLQISGER, FRLPENAK	3	IX
8	RS102_ARATH	40S ribosomal protein S10-2 OS = *Arabidopsis thaliana*	1	61	8.9	TYLNLPSEIVPATLK, TYLNLPSEIVPATLKK	2	V
8	RS193_ARATH	40S ribosomal protein S19-3 OS = *Arabidopsis thaliana*	1	60	15.4	DVSPHEFVK, ELAPYDPDWYYIR	2	V
1	CYF_AETCO	Apocytochrome f OS = *Aethionema cordifolium*	2	60	8.7	NILVIGPVPGQK, SNNTVYNATAGGIISK	2	II
8	UBIQP_ACECL	Polyubiquitin (Fragment) OS = *Acetabularia cliftonii*	1	60	8.7	IIFAGK, TLADYNIQK, ESTLHLVLR	3	V
8	RL40A_ARATH	Ubiquitin-60S ribosomal protein L40-1 OS = *Arabidopsis thaliana*	1	60	37.5	LIFAGK, TLADYNIQK, ESTLHLVLR	3	V
3	UREA_CANEN	Urease OS = *Canavalia ensiformis*	1	59	3	NYFLF, TIHTYHSEGAGGGHAPDIIK	2	I
8	RS13_PEA	40S ribosomal protein S13 OS = *Pisum sativum*	1	58	17.2	DSHGIAQVK, AHGLAPEIPEDLYHLIK	2	V
1,8	RAN_VICFA	GTP-binding nuclear protein Ran/TC4 OS = *Vicia faba*	2	58	13.1	HLTGEFEK, NLQYYEISAK	2	III/VI
7	PYRB_ARATH	Aspartate carbamoyltransferase, chloroplastic OS = *Arabidopsis thaliana*	1	57	5.4	GETLEDTIR, LGGEVLTTENAR	2	I
8	HSP11_CHERU	18.3 kDa class I heat shock protein OS = *Chenopodium rubrum*	1	52	18.6	FRLPENAK, IDWKETPEAHVFK	2	IX
5	CLAH1_ARATH	Clathrin heavy chain 1 OS = *Arabidopsis thaliana*	1	51	0.9	ILALK, SPEQVSAAVK	2	VI
6	PDI_RICCO	Protein disulfide-isomerase OS = *Ricinus communis*	1	49	4.2	FFNSPDAK, SEPIPEVNNEPVK	2	V
7	PDIA6_MEDSA	Probable protein disulfide-isomerase A6 OS *= Medicago sativa*	1	48	7.4	KLAPEYEK, YGVSGYPTIQWFPK	2	V
4	SUSY_MEDSA	Sucrose synthase OS = *Medicago sativa*	1	46	2.9	NITGLVEWYGK, SGFHIDPYHGDR	2	II
7	FKB62_ARATH	Peptidyl-prolyl cis-trans isomerase FKBP62 OS = *Arabidopsis thaliana*	1	42	4.4	SDGVEFTVK, FTLGQGQVIK	2	V
5	DLDH2_ARATH	Dihydrolipoyl dehydrogenase 2, mitochondrial OS = *Arabidopsis thaliana*	1	40	3.4	AAQLGLK, SLPGITIDEK	2	II
7	WIT2_ARATH	WPP domain-interacting tail-anchored protein 2 OS = *Arabidopsis thaliana*	1	39	3.3	ELELEK, AESGEAKIK	2	III
8	TBA_PRUDU	Tubulin alpha chain OS = *Prunus dulcis*	2	37	7.8	DVNAAVATIK, LVSQVISSLTASLR	2	VII
7	PER1B_ARMRU	Peroxidase C1B OS = *Armoracia rusticana*	1	35	5.7	VPLGR, MGNITPLTGTQGEIR	2	IX
7,(8,6)	YCF1_IPOPU	Putative membrane protein ycf1 OS = *Ipomoea purpurea*	3	35	1.3	ALILK, IVIEK, VIQEKER	3	X
6	RFS_ORYSJ	Galactinol--sucrose galactosyltransferase OS = *Oryza sativa* subsp. *Japonica*	1	27	2.8	VELAK, LMEEK	2	II
7	Y1497_ARATH	Probable receptor-like protein kinase At1g49730 OS = *Arabidopsis thaliana*	1	20	1.7	FLLAK, NLVALK	2	V

**^1^** Fraction corresponding to slice of the 1-D gel in which matches for the protein were found. Numbers in parenthesis indicate fractions where additional similar matches (see 2.) were found. ^2^ Number of protein matches of high taxonomical and sequence similarity grouped together with this match. (Match displayed was the top-scored one.) ^3^ MASCOT score. ^4^ I: metabolism, II: energy, III: cell growth/division, IV: transcription, V: protein synthesis/destination, VI: transporters, VII: cell structure, VIII: signal transduction, IX: disease/stress defense, and X: unclassified.

**Table 2:** List of top-scored non-redundant (nr) protein matches of the lotus embryo 1-D shotgun mass spectroscopy results, as matched to Green Plant proteome database (SwissProt 57.0).

Fractions ^1^	Protein Accession	Protein Description	Similar ^2^	Score ^3^	Cover (%)	Peptide sequences	Sig. Peptide Number	Func. Cat. ^4^
6,(5,2,1,7,3)	ENO1_HEVBR	Enolase 1 OS = *Hevea brasiliensis*	19	1125	42	TAIAK, YNQLLR, LTSEIGEK, DGGSDYLGK, AGWGVMASHR, MGAEVYHHLK, DGGSDYLGKGVSK, VQIVGDDLLVTNPK, VNQIGSVTESIEAVK, EAMKMGAEVYHHLK, AAVPSGASTGIYEALELR, LAMQEFMILPVGASSFK, SGETEDTFIADLSVGLATGQIK, YGQDATNVGDEGGFAPNIQENK, KYGQDATNVGDEGGFAPNIQENK, YGQDATNVGDEGGFAPNIQENKEGLELLK	16	II
4,(1,2,3,5)	HSP7C_PETHY	Heat shock cognate 70 kDa protein OS = *Petunia hybrida*	33	922	33.6	IEEVD, DISGNPR, NTTIPTKK, ITITNDKGR, DAGVIAGLNVMR, MVNHFVQEFK, NALENYAYNMR, MVNHFVQEFKR, TTPSYVGFTDTER, ARFEELNMDLFR, IINEPTAAAIAYGLDK, NQVAMNPINTVFDAK ATAGDTHLGGEDFDNR, NQVAMNPINTVFDAK, NAVVTVPAYFNDSQR, EQVFSTYSDNQPGVLIQVYEGER	16	IX
1,2,3,4,5,6,7	ACT_GOSHI	Actin OS = *Gossypium hirsutum*	14	903	50.7	DLTDALMK, AGFAGDDAPR, IKVVAPPER, GYSFTTTAER, HTGVMVGMGQK, EITALAPSSMK, DAYVGDEAQSK, AVFPSIVGRPR, IWHHTFYNELR, LDLAGRDLTDALMK, GYSFTTTAEREIVR, SYELPDGQVITIGAER, VAPEEHPVLLTEAPLNPK, VAPEEHPVLLTEAPLNPK, TTGIVLDSGDGVSHTVPIYEGYALPHAILR	15	VII
6,(1,2,3,7)	ACT12_SOLTU	Actin-100 (Fragment) OS = *Solanum tuberosum*	5	872	53.5	AGFAGDDAPR, IKVVAPPER, HTGVMVGMGQK, EITALAPSSMK, DAYVGDEAQSK, AVFPSIVGRPR, DAYVGDEAQSKR, GEYDESGPSIVHR, IWHHTFYNELR, SYELPDGQVITIGAER, LAYVALDYEQELETAK, YPIEHGIVSNWDDMEK, TTGIVLDSGDGVSHTVPIYEGYALPHAILR	13	VII
7,(8,2)	G3PC_ANTMA	Glyceraldehyde-3-phosphate dehydrogenase, cytosolic OS = *Antirrhinum majus*	20	749	43.6	VALQR, SSIFDAK, KATYEQIK, AAIKEESEGK, AGIALNDNFVK, DAPMFVVGVNEK, AASFNIIPSSTGAAK, VPTVDVSVVDLTVR, VPTVDVSVVDLTVRLEK, FGIVEGLMTTVHSITATQK, GILGYTEDDVVSTDFVGDSR, LTGMSFRVPTVDVSVVDLTVR, LKGILGYTEDDVVSTDFVGDSR, VINDRFGIVEGLMTTVHSITATQK	14	II
4,(1,5,2)	HSP83_IPONI	Heat shock protein 83 OS = *Ipomoea nil*	4	717	31	VIVTTK, VVVSDR, KLVSATK, AILFVPK, EMLQQNK, DVDGEQLGR, FESLTDKSK, APFDLFDTR, AVENSPFLER, LGIHEDSQNR, DIYYITGESK, LDAQPELFIR, RAPFDLFDTR, ADLVNNLGTIAR, ELISNASDALDK, KAVENSPFLER, HFSVEGQLEFK, GVVDSDDLPLNISR, ELISNASDALDKIR, SGDELTSLKDYVTR, KPEEITKEEYASFYK, HSEFISYPIYLWTEK, ITLFLKEDQLEYLEER	23	IX
6	ATPBM_MAIZE	ATP synthase subunit beta, mitochondrial OS = *Zea mays*	3	712	31.8	IGLFGGAGVGK, VVDLLAPYQR, TIAMDGTEGLVR, AHGGFSVFAGVGER, VGLTGLTVAEHFR, VLNTGSPITVPVGR, TVLIMELINNVAK, FTQANSEVSALLGR, QISELGIYPAVDPLDSTSR, EAPAFVEQATEQQILVTGIK, IPSAVGYQPTLATDLGGLQER	11	I
4,(5,2,1,3)	HSP7E_SPIOL	Chloroplast envelope membrane 70 kDa heat shock-related protein OS = *Spinacia oleracea*	5	631	31.2	NTTIPTKK, LSKEEIEK, TRDNNLLGK, DAGVISGLNVMR, EIAEAYLGSTVK, NALENYAYNMR, TTPSYVAFTDSER, IINEPTAAAIAYGLDK, ATAGDTHLGGEDFDNR, NQVAMNPINTVFDAK, NAVVTVPAYFNDSQR, EQVFSTYSDNQPGVLIQVYEGER	12	IX
4,(2,1,5)	HSP81_ORYSI	Heat shock protein 81-1 OS = *Oryza sativa* subsp. *Indica*	9	610	35.1	NLVKK, VVVTTK, IAELLR, KLVSATK, EMLQQNK, FESLTDKSK, APFDLFDTR, DSSMAGYMSSK, RAPFDLFDTR, KAVENSPFLEK, SDLVNNLGTIAR, HFSVEGQLEFK, EVSHEWSLVNK, GIVDSEDLPLNISR, SLTNDWEEHLAVK, SGDELTSLKDYVTR, LDAQPELFIHIVPDK, HSEFISYPISLWTEK, KPEEITKEEYAAFYK, MKEGQNDIYYITGESK, KHSEFISYPISLWTEK	21	IX
6,(3,1,2)	TBB_HORVU	Tubulin beta chain OS = *Hordeum vulgare*	21	583	39.1	YLTASAMFR, IREEYPDR, LAVNLIPFPR, VSEQFTAMFR, YTGTSDLQLER, MMLTFSVFPSPK, EVDEQMINVQNK, LHFFMVGFAPLTSR, AVLMDLEPGTMDSVR, LHFFMVGFAPLTSR, NSSYFVEWIPNNVK, ALTVPELTQQMWDAK, GHYTEGAELIDSVLDVVRK, TGPYGQIFRPDNFVFGQSGAGNNWAK	14	VII
6,(1,7,3,4,2)	EF1A_TOBAC	Elongation factor 1-alpha OS = *Nicotiana tabacum*	17	574	38.5	YDEIVK, GFVASNSK, EVSSYLK, QTVAVGVIK, EVSSYLKK, RGFVASNSK, LPLQDVYK, ARYDEIVK, IGGIGTVPVGR, STNLDWYK, STTTGHLIYK, EHALLAFTLGVK, GFVASNSKDDPAK, YYCTVIDAPGHR, YDEIVKEVSSYLK, YYCTVIDAPGHRDFIK, MIPTKPMVVETFSEYPPLGR, NMITGTSQADCAVLIIDSTTGGFEAGISK	18	V
4,(1,2,5,4)	HS901_ARATH	Heat shock protein 90-1 OS = *Arabidopsis thaliana*	6	539	26.4	VVVTTKVVVTTK, VVVSDR, KLVSATK, AILFVPK, FESLTDKSK, APFDLFDTR, AVENSPFLER, LGIHEDSQNR, DSSMSGYMSSK, RAPFDLFDTR, ADLVNNLGTIAR, KAVENSPFLER, HFSVEGQLEFK, TLSIIDSGIGMTK, GVVDSDDLPLNISR, KPEEITKEEYAAFYK, HSEFISYPIYLWTEK	17	IX
4,(2,1,3)	METE_ARATH	5-methyltetrahydropteroyltriglutamate-homocysteine methyltransferase OS = *Arabidopsis thaliana*	12	536	20.9	AAAALK, VVEVNALAK, SWLAFAAQK, AVNEYKEAK, YLFAGVVDGR, SDEKLLSVFR, FALESFWDGK, GNASVPAMEMTK, YGAGIGPGVYDIHSPR, GMLTGPVTILNWSFVR	10	I
4	ENPL_CATRO	Endoplasmin homolog OS = *Catharanthus roseus*	3	472	12.6	NLGTIAK, FWNEFGK, YGWSSNMER, ELISNASDALDK, IMQSQTLSDASK, GLVDSDTLPLNVSR, ELISNASDALDKIR, VFISDEFDELLPK, RVFISDEFDELLPK, LMDIIINSLYSNKDIFLR	10	IX
4,(5,2)	BIP4_TOBAC	Luminal-binding protein 4 OS = *Nicotiana tabacum*	6	454	21	LIGEAAK, NTVIPTKK, IMEYFIK, LSQEEIER, ITITNDKGR, ALSSQHQVR, EAEEFAEEDKK, IVNKDGKPYIQVK, ARFEELNNDLFR, IINEPTAAAIAYGLDK, IKDAVVTVPAYFNDAQR	11	IX
4,(1,3,2,7)	EF2_BETVU	Elongation factor 2 OS = *Beta vulgaris*	6	454	16.6	DLYVK, VASDLPK, GGGQIIPTAR, MIPASDKGR, IRPVLTVNK, EGALAEENMR, NMSVIAHVDHGK, FGVDESKMMER, VFYASQLTAKPR, LWGENFFDPATK, IRPVLTVNKMDR, RVFYASQLTAKPR, GHVFEEMQRPGTPLYNIK, RGHVFEEMQRPGTPLYNIK	15	V
4,(1,2,3)	HSP82_MAIZE	Heat shock protein 82 OS = *Zea mays*	5	429	16.1	VVVSDR, KLVSATK, APFDLFDTR, AVENSPFLER, LGIHEDSQNR, RAPFDLFDTR, SDLVNNLGTIAR, ELISNASDALDK, KAVENSPFLER, HFSVEGQLEFK, GVVDSDDLPLNISR, ELISNASDALDKIR, HSEFISYPIYLWTEK	13	IX
6,(2,3)	IF4A1_ORYSJ	Eukaryotic initiation factor 4A-1 OS = *Oryza sativa* subsp. *Japonica*	4	418	31.4	ALGDYLGVK, ELAQQIEK, KGVAINFVTR, VLITTDLLAR, QSLRPDYIK, RDELTLEGIK, GLDVIQQAQSGTGK, GIYAYGFEKPSAIQQR, GFKDQIYDIFQLLPSK	9	V
3,(2,1)	CAPPC_FLATR	Phosphoenolpyruvate carboxylase 2 OS = *Flaveria trinervia*	32	417	18	GIAAGMQNTG, MNIGSRPSK, VILGDVRDK, KPSGGIESLR, LSAAWQLYK, SPEEVFDALK, RPLFGPDLPK, TPPTPQDEMR, QVSTFGLSLVR, VTIDLVEMVFAK, AGMSYFHETIWK, AIPWIFAWTQTR, VPYNAPLIQFSSWMGGDRDGNPR	13	I
6,(2)	ENO2_ARATH	Bifunctional enolase 2/transcriptional activator OS = *Arabidopsis thaliana*	2	391	33.3	YNQLLR, DGGSDYLGK, ISGDALKDLYK, DGGSDYLGKGVSK, VNQIGSVTESIEAVK, IVLPVPAFNVINGGSHAGNK, SGETEDTFIADLAVGLSTGQIK, YGQDATNVGDEGGFAPNIQENK, KYGQDATNVGDEGGFAPNIQENK, YGQDATNVGDEGGFAPNIQENKEGLELLK	10	IV
5,(2)	CH61_CUCMA	Chaperonin CPN60-1, mitochondrial OS = *Cucurbita maxima*	6	387	33.33	ISSINAVVK, VTDALNATK, VTKDGVTVAK, KISSINAVVK, IGGASEAEVGEK, IGVQIIQNALK, IGGASEAEVGEKK, GYISPYFITNQK, AAVEEGIVPGGGVALLYASK, TPVHTIASNAGVEGAVVVGK	10	IX
4	HSP7L_ARATH	Heat shock 70 kDa protein 12 OS = *Arabidopsis thaliana*	1	382	16.8	NTVIPTKK, IMEYFIK, ALSSQHQVR, EAEEFAEEDKK, ARFEELNNDLFR, ARFEELNNDLFR, IINEPTAAAIAYGLDK, IKDAVVTVPAYFNDAQR	8	IX
2,(1)	CLAH1_ARATH	Clathrin heavy chain 1 OS = *Arabidopsis thaliana*	4	374	10.3	TVDNDLALK, SPEQVSAAVK, VANVELYYK, DPTLAVVAYR, FQELFAQTK, VEEDAVWSQVAK, GNLPGAENLVVQR, EGLVSDAIESFIR, GNMQLFSVDQQR, KNLLENWLAEDK, RGNLPGAENLVVQR, QLIDQVVSTALPESK, YKEAAELAAESPQGILR	13	VI
6,(2,5,1)	ATPAM_PEA	ATP synthase subunit alpha, mitochondrial OS = *Pisum sativum*	1	364	29.4	VVSVGDGIAR, TGSIVDVPAGK, AAELTTLLESR, VVDALGVPIDGR, TAIAIDTILNQK, KSVHEPMQTGLK, GIRPAINVGLSVSR, EAFPGDVFYLHSR, ITNFYTNFQVDEIGR, LTEVLKQPQYAPLPIEK, EVAAFAQFGSDLDAATQALLNR	11	I
5	RUBB_PEA	RuBisCO large subunit-binding protein subunit beta, chloroplastic OS = *Pisum sativum*	2	357	23.98	IAALK, VVLTK, NVVLESK, VEDALNATK, IVNDGVTVAK, KGVVTLEEGK, LADLVGVTLGPK, GYISPYFVTDSEK, EVELEDPVENIGAK, TNDLAGDGTTTSVVLAQGLIAEGVK, IVNDGVTVAKEVELEDPVENIGAK	11	I
5,(2)	PGMC_PEA	Phosphoglucomutase, cytoplasmic OS = *Pisum sativum*	5	333	41.16	YLFEDGSR, FFEVPTGWK, LSGTGSEGATIR, SMPTSAALDVVAK, YDYENVDAGAAK	5	II
6,(2)	SAHH_MESCR	Adenosylhomocysteinase OS = *Mesembryanthemum crystallinum*	6	330	11.1	ATDVMIAGK, HSLPDGLMR, ITIKPQTDR, TEFGPSQPFK, LVGVSEETTTGVK, TEFGPSQPFKGAK, LVGVSEETTTGVKR	7	I
7	PGKY_TOBAC	Phosphoglycerate kinase, cytosolic OS = *Nicotiana tabacum*	7	302	21.7	LAELSGK, YSLKPLVPR, YLKPAVAGFLMQK, GVSLLLPTDVVIADK, GVTTIIGGGDSVAAVEK, LASLADLYVNDAFGTAHR, KLASLADLYVNDAFGTAHR	7	II
4	SUSY_SOYBN	Sucrose synthase OS = *Glycine max*	12	295	13.4	YLEMFYALK, VVHGIDVFDPK, NITGLVEWYGK, ELVNLVVVAGDR, LLPDAVGTTCGQR, SGFHIDPYHGDR, LGVTQCTIAHALEK	7	II
5	CPNB3_ARATH	Chaperonin 60 subunit beta 3, chloroplastic OS = *Arabidopsis thaliana*	1	292	25.35	VVLTK, NVVLESK, VEDALNATK, KGVVTLEEGK, LADLVGVTLGPK, GYISPYFVTDSEK, EVELEDPVENIGAK, TNDLAGDGTTTSVVLAQGLIAEGVK	8	IX
7,(8)	MDHC2_ARATH	Malate dehydrogenase, cytoplasmic 2 OS = *Arabidopsis thaliana*	2	288	23.2	GAAIIK, NVSIYK, SQASALEK, EFAPSIPEK, MELVDAAFPLLK, VLVVANPANTNALILK, VLVTGAAGQIGYALVPMIAR	7	II
8,(1)	1433E_TOBAC	14-3-3-like protein E OS = *Nicotiana tabacum*	19	284	29.8	NVIGAR, VFYLK, YLAEFK, MKGDYHR, NLLSVAYK, IISSIEQK, TVDVEELTVEER, IISSIEQKEESR, SAQDIALAELAPTHPIR	9	VIII
5	VATA_GOSHI	V-type proton ATPase catalytic subunit A OS = *Gossypium hirsutum*	2	272	49.1	SGDVYIPR, TVISQALSK, LAADTPLLTGQR, LAEMPADSGYPAYLAAR, LTTFEDSEKESEYGYVR, LVSQKFEDPAEGEEALVAK	6	VI
4,(3,2,1)	CD48A_ARATH	Cell division control protein 48 homolog A OS = *Arabidopsis thaliana*	3	265	18.5	TLLAK, KGDLFLVR, RSVSDADIR, DFSTAILER, LAEDVDLER, GILLYGPPGSGK, LAGESESNLRK, IVSQLLTLMDGLK, ELVELPLRHPQLFK, NAPSIIFIDEIDSIAPK	10	III/IV
7	ALF_CICAR	Fructose-bisphosphate aldolase, cytoplasmic isozyme OS = *Cicer arietinum*	1	245	11.1	GILAADESTGTIGK, GILAADESTGTIGKR, YHDELIANAAYIGTPGK	3	II
7	MDHM_CITLA	Malate dehydrogenase, mitochondrial OS = *Citrullus lanatus*	2	235	17.6	LFGVTTLDVVR, TQDGGTEVVEAK, DDLFNINAGIVK, RTQDGGTEVVEAK, VAVLGAAGGIGQPLALLMK	5	II
6,7	ACT5_ARATH	Putative actin-5 OS = *Arabidopsis thaliana*	1	213	20.4	AGFAGDDAPR, IKVVAPPER, IWHHTFYNELR, TTGIVLDSGDGVSHTVPIYEGYALPHAILR	4	VII
6	UGPA_MUSAC	UTP--glucose-1-phosphate uridylyltransferase OS = *Musa acuminata*	3	199	18	VANFLSR, GGTLISYEGR, VLQLETAAGAAIR, FFDHAIGINVPR, LQSAVAELNQISENEK	5	II
8	ADT1_GOSHI	ADP, ATP carrier protein 1, mitochondrial OS = *Gossypium hirsutum*	2	194	8.8	SSLDAFSQILK, LLIQNQDEMIK, YFPTQALNFAFK	3	VI
8	RAN_VICFA	GTP-binding nuclear protein Ran/TC4 OS = *Vicia faba*	1	193	26.2	NVPTWHR, HLTGEFEK, AKQVTFHR, LVIVGDGGTGK, NLQYYEISAK, SNYNFEKPFLYLAR	6	VIII
3	CLPB1_ARATH	Chaperone protein ClpB1 OS = *Arabidopsis thaliana*	2	192	11.1	TAVVEGLAQR, YRGEFEER, TKNNPVLIGEPGVGK, KVESASGDTNFQALK, VQLDSQPEEIDNLER, LIGAPPGYVGHEEGGQLTEAVR	6	IX
5	CPNA1_ARATH	Chaperonin 60 subunit alpha 1, chloroplastic OS = *Arabidopsis thaliana*	1	189	82.82	VVNDGVTIAR, NVVLDEFGSPK, VGAATETELEDR	3	IX
3	ACOC_CUCMA	Aconitate hydratase, cytoplasmic OS = *Cucurbita maxima*	6	187	9.2	NFEGR, ILLESAIR, STYESITK, DFNSYGSR, RGNDEVMAR, TSLAPGSGVVTK, ATIANMSPEYGATMGFFPVDHVTLQYLK	7	II
3	SYA_ARATH	Alanine--tRNA ligase OS = *Arabidopsis thaliana*	1	181	5.4	LTSVLQNK, HVDTGMGFER, ESDGSLKPLPAK, AFALLSEEGIAK, AVFGEVYPDPVR	5	IV
6,(7)	PRS6A_SOLLC	26S protease regulatory subunit 6A homolog OS = *Solanum lycopersicum*	4	170	8.5	IIKEELQR, GVLLYGPPGTGK, LAGPQLVQMFIGDGAK	3	I
7	AATM_LUPAN	Aspartate aminotransferase P2, mitochondrial (Fragment) OS = *Lupinus angustifolius*	1	170	10.8	IADVIQEK, NLGLYAER, LNLGVGAYR, ISLAGLSLAK, VATVQGLSGTGSLR	5	I
6	UGDH_SOYBN	UDP-glucose 6-dehydrogenase OS = *Glycine max*	1	168	6.5	IAILGFAFK, LAANAFLAQR, AADLTYWESAAR	3	II
2,8	ANX4_FRAAN	Annexin-like protein RJ4 OS = *Fragaria ananassa*	1	163	9.6	VGTDEDALTR, LLVALVTAYR	2	IX
8	RS4_GOSHI	40S ribosomal protein S4 OS = *Gossypium hirsutum*	4	163	22.9	LSIIEEAR, LGNVFTIGK, GIPYLNTYDGR, LGGAFAPKPSSGPHK, TDKTYPAGFMDVVSIPK	5	V
7,(8)	RSSA_SOYBN	40S ribosomal protein SA OS = *Glycine max*	2	161	19	LLILTDPR, YVDIGIPANNK, HTPGTFTNQLQTSFSEPR, VIVAIENPQDIIVQSARPYGQR	4	V
8	RAA1D_ARATH	Ras-related protein RABA1d OS = *Arabidopsis thaliana*	9	161	26.6	AITSAYYR, VVLIGDSGVGK, STIGVEFATR, HSTFENVER, AQIWDTAGQER	5	VIII
8	RS18_ARATH	40S ribosomal protein S18 OS = *Arabidopsis thaliana*	1	155	23.7	LRDDLER, VLNTNVDGK, IPDWFLNR, YSQVVSNALDMK	4	V
1	AVP_VIGRR	Pyrophosphate-energized vacuolar membrane proton pump OS = *Vigna radiata* var. *Radiata*	1	151	9.5	TDALDAAGNTTAAIGK, AAVIGDTIGDPLKDTSGPSLNILIK	2	VI
5	ILV5_ARATH	Ketol-acid reductoisomerase, chloroplastic OS = *Arabidopsis thaliana*	1	150	27.45	SDIVVK, SVVLAGR, QIGVIGWGSQGPAQAQNLR	3	I
7	AATC_DAUCA	Aspartate aminotransferase, cytoplasmic OS = *Daucus carota*	2	149	10.4	ISMAGLSSR, LNLGVGAYR, LIFGADSPAIQENR	3	I
8	RL13_TOBAC	60S ribosomal protein L13 OS = *Nicotiana tabacum*	1	149	23.3	GFSLEELK, TWFNQPAR, SLEGLQTNVQR, KLAPTIGIAVDHR	4	V
5	ACLB1_ORYSJ	ATP-citrate synthase beta chain protein 1 OS = *Oryza sativa* subsp. *Japonica*	1	148	35.6	FNNIPQVK, FGGAIDDAAR, SEVQFGHAGAK, SIGLIGHTFDQKR, VVAIIAEGVPESDTK	5	I
2,(3)	COPA1_ARATH	Coatomer subunit alpha-1 OS = *Arabidopsis thaliana*	3	147	4.4	VWDIGALR, YVLEGHDR, AWEVDTLR, VVIFDLQQR, TLDVPIYITK, QDIIVSNSEDK	6	VI
6	CATA2_RICCO	Catalase isozyme 2 OS = *Ricinus communis*	2	146	9.3	FSTVIHER, APGVQTPVIVR, EGNFDIVGNNFPVFFIR	3	IX
8	RAN3_ORYSI	GTP-binding nuclear protein Ran-3 OS = *Oryza sativa* subsp. *Indica*	1	145	14.2	HITGEFEK, NLQYYEISAK, SNYNFEKPFLYLAR	3	III/VI
6	MDAR_SOLLC	Monodehydroascorbate reductase OS = *Solanum lycopersicum*	1	139	12.5	AYLFPEGAAR, LSDFGVQGADSK, IVGAFLESGSPEENKAIAK	3	IX
6,(1)	RL3_ORYSJ	60S ribosomal protein L3 OS = *Oryza sativa* subsp. *Japonica*	4	138	9.3	VIAHTQIR, HGSLGFLPR, LALEEIKLK, GKGYEGVVTR	4	V
5	PMGI_RICCO	2,3-bisphosphoglycerate-independent phosphoglycerate mutase OS = *Ricinus communis*	4	136	43.5	ARDAILSGK, LVDLALASGK, LDQLQLLLK, AHGTAVGLPTEDDMGNSEVGHNALGAGR	4	II
5	RUBA_RICCO	RuBisCO large subunit-binding protein subunit alpha (Fragment) OS = *Ricinus communis*	2	134	71.19	NVVLDEFGSPK, VGAATETELEDR, LGLLSVTSGANPVSIK	3	I
7	RL4_PRUAR	60S ribosomal protein L4 OS = *Prunus armeniaca*	1	134	14.5	AGHQTSAESWGTGR, YAVVSAIAASAVPSLVLAR	2	V
5	G6PI_SPIOL	Glucose-6-phosphate isomerase, cytosolic OS = *Spinacia oleracea*	2	131	27	SQQPVYLK, FLANVDPIDVAK, TFTTAETMLNAR	3	II
8	RS8_MAIZE	40S ribosomal protein S8 OS = *Zea mays*	1	130	21.7	LDTGNYSWGSEAVTR, ILDVVYNASNNELVR	2	V
8	RL11_MEDSA	60S ribosomal protein L11 OS = *Medicago sativa*	1	129	17.7	YEGVILNK, AMQLLESGLK, VLEQLSGQTPVFSK	3	V
8,(7)	H4_ARATH	Histone H4 OS = *Arabidopsis thaliana*	2	128	46.6	TLYGFGG, IFLENVIR, DAVTYTEHAR, ISGLIYEETR	4	VII
5	TCPE_ARATH	T-complex protein 1 subunit epsilon OS = *Arabidopsis thaliana*	1	123	73.41	IAEGYEMASR, QQQILLATQVVK	2	V
5	TCPA_ARATH	T-complex protein 1 subunit alpha OS = *Arabidopsis thaliana*	1	119	71.23	YFVEAGAIAVR, NKIHPTSIISGYR	2	V
4	TKTC_SPIOL	Transketolase, chloroplastic OS = *Spinacia oleracea*	4	118	6.1	FLAIDAVEK, ALPTYTPETPGDATR, VIPGLLGGSADLASSNMTLLK	3	II
8	TPIS_MAIZE	Triosephosphate isomerase, cytosolic OS = *Zea mays*	1	117	11.9	FFVGGNWK, VAYALSQGLK, VIACVGETLEQR	3	VII
8	PROF3_ARATH	Profilin-3 OS = *Arabidopsis thaliana*	2	117	17.2	LGDYLLEQGL, YMVIQGEPGAVIR	2	VII
8	RS92_ARATH	40S ribosomal protein S9-2 OS = *Arabidopsis thaliana*	1	115	19.3	LVGEYGLR, ERLDAELK, RPYEKER, RLQTIVFK, IFEGEALLR	5	V
6	VATB1_ARATH	V-type proton ATPase subunit B1 OS = *Arabidopsis thaliana*	1	115	10.5	YQEIVNIR, TVSGVAGPLVILDK, QIYPPINVLPSLSR	3	VI
6	ERF1X_ARATH	Eukaryotic peptide chain release factor subunit 1-1 OS = *Arabidopsis thaliana*	1	113	9.9	GFGGIGGILR, QSVLGAITSAQQR	2	V
5	HSP7M_PHAVU	Heat shock 70 kDa protein, mitochondrial OS = *Phaseolus vulgaris*	3	111	24.87	HLNITLTR, VIENSEGAR, TTPSVVAFNQK, SSGGLSEDEIEK	4	IX
8	ANXD1_ARATH	Annexin D1 OS = *Arabidopsis thaliana*	1	110	5	AQINATFNR, SKAQINATFNR	2	IX
8	RS16_FRIAG	40S ribosomal protein S16 OS = *Fritillaria agrestis*	3	109	13.8	ALVAYYQK, AFEPILLLGR, YKAFEPILLLGR	3	V
8	RS5_CICAR	40S ribosomal protein S5 (Fragment) OS = *Cicer arietinum*	1	108	15.7	IGSAGVVRR, GSSNSYAIK, VNQAIYLLTTGAR	3	V
8	ARF_VIGUN	ADP-ribosylation factor OS = *Vigna unguiculata*	1	104	28.7	ILMVGLDAAGK, NISFTVWDVGGQDK	2	VIII
4	SYGM1_ARATH	Glycine--tRNA ligase 1, mitochondrial OS = *Arabidopsis thaliana*	1	103	5.1	LFYIPSFK, VFTPSVIEPSFGIGR	2	IV/VI
7	RGP1_ORYSJ	UDP-arabinopyranose mutase 1 OS = *Oryza sativa* subsp. *Japonica*	1	101	6.9	ILGPK, ASNPFVNLK, ASNPFVNLKK, YVDAVMTVPK	4	I
3	HSP7O_ARATH	Heat shock 70 kDa protein 14 OS = *Arabidopsis thaliana*	1	101	5.1	ILSHAFDR, NAVESYVYDMR, AVLDAATIAGLHPLR	3	IX
8	RS15A_DAUCA	40S ribosomal protein S15a OS = *Daucus carota*	1	100	29.2	VSVLNDALK, HGYIGEFEYVDDHR	2	V
8	RS61_ARATH	40S ribosomal protein S6-1 OS = *Arabidopsis thaliana*	2	100	18	LVTPLTLQR, KGENDLPGLTDTEKPR, ISQEVSGDALGEEFKGYVFK	3	V
6	ACCC2_POPTR	Biotin carboxylase 2, chloroplastic OS = *Populus trichocarpa*	1	97	7.8	LLEEAPSPALTPELR, ALDDTVITGVPTTIDYHK	2	I
8	RLA2_PARAR	60S acidic ribosomal protein P2 OS = *Parthenium argentatum*	1	97	10.5	DITELIASGR, GKDITELIASGR	2	V
8	RS33_ARATH	40S ribosomal protein S3-3 OS = *Arabidopsis thaliana*	3	96	10.9	ELAEDGYSGVEVR, FKFPQDSVELYAEK	2	V
5	KPYC_SOYBN	Pyruvate kinase, cytosolic isozyme OS = *Glycine max*	1	94	55.66	KGSDLVNVR, STPLPMSPLESLASSAVR	2	II
7	GMD1_ARATH	GDP-mannose 4,6 dehydratase 1 OS = *Arabidopsis thaliana*	2	93	5	RGENFVTR, LFLGNIQASR	2	II
4	HSP7S_SPIOL	Stromal 70 kDa heat shock-related protein, chloroplastic (Fragment) OS = *Spinacia oleracea*	2	93	5.3	HIETTLTR, IINEPTAASLAYGFEK	2	IX
6	EF1G2_ORYSJ	Elongation factor 1-gamma 2 OS = *Oryza sativa* subsp. *Japonica*	3	93	16.7	EVAIK, LYSNTK, NPLDLLPPSK, MILDEWKR, SFTSEFPHVER	5	V
8	RLA0_LUPLU	60S acidic ribosomal protein P0 OS = *Lupinus luteus*	1	90	9.9	EYLKDPSK, VGSSEAALLAK	2	V
7	GCST_PEA	Aminomethyltransferase, mitochondrial OS = *Pisum sativum*	1	89	8.3	GGAIDDSVITK, TGYTGEDGFEISVPSEHGVELAK	2	I
8	APX1_ORYSJ	L-ascorbate peroxidase 1, cytosolic OS = *Oryza sativa* subsp. *Japonica*	1	89	15.2	TGGPFGTMK, LSELGFADA, ALLSDPAFRPLVEK	3	IX
8	RS193_ARATH	40S ribosomal protein S19-3 OS = *Arabidopsis thaliana*	1	88	23.1	AYAAHLKR, TVKDVSPHEFVK, ELAPYDPDWYYIR	3	V
1,(2)	RPN1A_ARATH	26S proteasome non-ATPase regulatory subunit 2 1A OS = *Arabidopsis thaliana*	3	87	5.5	VGQAVDVVGQAGRPK, NLAGEIAQEYTKR	2	I
4,(3)	PPDK_FLABR	Pyruvate, phosphate dikinase, chloroplastic OS = *Flaveria browni*i	8	87	2.9	SDFEGIFR, AALIADEIAK, AMDGLPVTIR	3	II
8	RS13_PEA	40S ribosomal protein S13 OS = *Pisum sativum*	1	87	25.8	DSHGIAQVK, GLTPSQIGVILR, KGLTPSQIGVILR, AHGLAPEIPEDLYHLIK	4	V
8	RS14_CHLRE	40S ribosomal protein S14 OS = *Chlamydomonas reinhardtii*	1	85	18.3	TPGPGAQSALR, IEDVTPIPTDSTRR	2	V
6	VATB2_GOSHI	V-type proton ATPase subunit B 2 (Fragment) OS = *Gossypium hirsutum*	1	84	10.1	FVTQGAYDTR, QIYPPINVLPSLSR	2	VI
8,(5)	CYPH_MAIZE	Peptidyl-prolyl cis-trans isomerase OS = *Zea mays*	3	83	16.3	SGKPLHYK, VFFDMTVGGAPAGR	2	V
8	NDK1_ARATH	Nucleoside diphosphate kinase 1 OS = *Arabidopsis thaliana*	1	82	9.4	NVIHGSDSVESAR, NVIHGSDSVESARK	2	I
7	GLN11_ORYSJ	Glutamine synthetase cytosolic isozyme 1-1 OS = *Oryza sativa* subsp. *Japonica*	1	79	7.6	DIVDSHYK, HKEHISAYGEGNER	2	I
7	SERC_SPIOL	Phosphoserine aminotransferase, chloroplastic OS = *Spinacia oleracea*	1	79	5.3	FGLIYAGAQK, NVGPSGVTIVIVR	2	I
7	PDI21_ARATH	Protein disulfide-isomerase like 2-1 OS = *Arabidopsis thaliana*	1	78	8.9	KLAPEYEK, YGVSGFPTLK, YGVSGYPTIQWFPK	3	V
8	RS254_ARATH	40S ribosomal protein S25-4 OS = *Arabidopsis thaliana*	2	76	29.6	LITPSILSDR, MVAAHSSQQIYTR	2	V
6	IDHC_TOBAC	Isocitrate dehydrogenase [NADP] OS = *Nicotiana tabacum*	2	75	7.5	HAFGDQYR, DLALIIHGSK, TIEAEAAHGTVTR	3	II
6	OPD22_ARATH	Dihydrolipoyllysine-residue acetyltransferase component 2 of pyruvate dehydrogenase complex, mitochondrial OS = *Arabidopsis thaliana*	1	73	3.9	ISVNDLVIK, VIDGAIGAEWLK	2	II
8	RL40A_ARATH	Ubiquitin-60S ribosomal protein L40-1 OS = *Arabidopsis thaliana*	1	70	45.3	ESTLHLVLR, TITLEVESSDTIDNVK	2	V
8	RL24_PRUAV	60S ribosomal protein L24 OS = *Prunus avium*	1	69	7	SIVGATLEVIQK, SIVGATLEVIQKR	2	V
7,(5)	SAPK6_ORYSJ	Serine/threonine-protein kinase SAPK6 OS = *Oryza sativa* subsp. *Japonica*	2	68	7.4	DIGSGNFGVAR, STVGTPAYIAPEVLSR	2	III
6	GME2_ORYSJ	GDP-mannose 3,5-epimerase 2 OS = *Oryza sativa* subsp. *Japonica*	1	67	7	NSDNTLIKEK, ISITGAGGFIASHIAR	2	II
8	EF1D1_ORYSJ	Elongation factor 1-delta 1 OS = *Oryza sativa* subsp. *Japonica*	2	66	7.9	LVPVGYGIK, KLDEYLLTR	2	V
8	IF5A1_ARATH	Eukaryotic translation initiation factor 5A-1 OS = *Arabidopsis thaliana*	1	64	12	VVEVSTSK, TYPQQAGTIR, TYPQQAGTIRK	3	V
8	H2B11_ARATH	Histone H2B.11 OS = *Arabidopsis thaliana*	1	62	30	LVLPGELAK, QVHPDIGISSK, YNKKPTITSR	3	VII
8	PSA3_ARATH	Proteasome subunit alpha type-3 OS = *Arabidopsis thaliana*	1	60	7.6	VFQIEYAAK, VPDDLLEEAK	2	I
7	AAT3_ARATH	Aspartate aminotransferase, chloroplastic OS = *Arabidopsis thaliana*	1	60	4.9	LNLGVGAYR, TEEGKPLVLNVVR	2	I
1	PDR4_ORYSJ	Pleiotropic drug resistance protein 4 OS = *Oryza sativa s*ubsp. *Japonica*	1	60	1.5	TTLLLALAGK, VTTGEMLVGPAR	2	IX
2,(8)	UBQ12_ARATH	Polyubiquitin 12 OS = *Arabidopsis thaliana*	3	60	23.9	MQIFLKTLTGK, IQDKEGIPPDQQR, TITLEVESSDTIDNVK	3	V
2,(1)	UBIQ_AVESA	Ubiquitin OS = *Avena sativa*	1	60	57.9	TLADYNIQK, IQDKEGIPPDQQR, TITLEVESSDTIDNVK	3	V
5,(1)	DIM_PEA	Delta(24)-sterol reductase OS = *Pisum sativum*	2	59	45.72	NILDIDKER, SDLEAPLRPK	2	I
8	HSP11_PEA	18.1 kDa class I heat shock protein OS = *Pisum sativum*	2	56	8.9	SIEISG, VLQISGER	2	IX
6	MPPA_SOLTU	Mitochondrial-processing peptidase subunit alpha OS = *Solanum tuberosum*	1	52	3.4	QLLTYGER, MVASEDIGR	2	I
8	RL17_MAIZE	60S ribosomal protein L17 OS = *Zea mays*	1	52	10.5	NAESNADVK, YLEDVIAHK	2	V
8	RL51_ARATH	60S ribosomal protein L5-1 OS = *Arabidopsis thaliana*	2	52	9.3	VFGALK, KLTYEER, GALDGGLDIPHSDKR	3	V
3	PHSL1_SOLTU	Alpha-1,4 glucan phosphorylase L-1 isozyme, chloroplastic/amyloplastic OS = *Solanum tuberosum*	1	50	1.8	NDVSYPIK, AFATYVQAK	2	II
6	PRS4A_ARATH	26S proteasome regulatory subunit 4 homolog A OS = *Arabidopsis thaliana*	1	49	9.5	VVGSELIQK, GVILYGEPGTGK	2	II
8	YPTC1_CHLRE	GTP-binding protein YPTC1 OS = *Chlamydomonas reinhardtii*	2	49	17.2	TITSSYYR, LLLIGDSGVGK	2	III/VI
4	HSP7G_ARATH	Heat shock 70 kDa protein 7, chloroplastic OS = *Arabidopsis thaliana*	1	47	9.5	HIETTLTR, TTPSVVAYTK, QAVVNPENTFFSVKR	3	IX
8	SODM_HEVBR	Superoxide dismutase [Mn], mitochondrial OS = *Hevea brasiliensis*	1	46	11.2	HHQTYITNYNK, LVVETTANQDPLVTK	2	IX
5	CALX2_ARATH	Calnexin homolog 2 OS = *Arabidopsis thaliana*	2	45	30.3	NPAYK, SEGHDDYGLLVSEK	2	V
8	RS30_ARATH	40S ribosomal protein S30 OS = *Arabidopsis thaliana*	1	44	30.6	GKVHGSLAR, FVTAVVGFGK	2	V
8	RL7A1_ARATH	60S ribosomal protein L7a-1 OS = *Arabidopsis thaliana*	1	44	9.3	TLDKNLATSLFK, LKVPPALNQFTK	2	V
7	METK4_POPTR	S-adenosylmethionine synthase 4 OS = *Populus trichocarpa*	1	42	12.3	FVIGGPHGDAGLTGR, VLVNIEQQSPDIAQGVHGHLTK	2	I
8	RL18A_CASSA	60S ribosomal protein L18a OS = *Castanea sativa*	1	41	9.6	ASRPNLFM, FHQYQVVGR	2	V
7	EFTM_ARATH	Elongation factor Tu, mitochondrial OS = *Arabidopsis thaliana*	1	41	9.7	QAILK, VLAEEGKAK, GITIATAHVEYETAKR	3	V
1	CALSB_ARATH	Callose synthase 11 OS = *Arabidopsis thaliana*	1	38	1.5	ILFNEAFSR, LGEGKPENQNHALIFTR	2	IX
3	APBLB_ARATH	Beta-adaptin-like protein B OS = *Arabidopsis thaliana*	1	27	2	EAENIVER, DSQDPNPLIR	2	VII

^1^ Fraction corresponding to slice of the 1-D gel in which matches for the protein were found. Numbers in parenthesis indicate fractions where additional similar matches (see ^2^) were found. ^2^ Number of protein matches of high taxonomical and sequence similarity grouped together with this match. (Match displayed was the top-scored one.) ^3^ MASCOT score. ^4^ I: metabolism, II: energy, III: cell growth/division, IV: transcription, V: protein synthesis/destination, VI: transporters, VII: cell structure, VIII: signal transduction, IX: disease/stress defense, and X: unclassified

### 3.4. Comparative Analysis of Lotus Seed (Immature Endosperm, Mature Endosperm, and Embryo) Proteins

**Figure 3:**
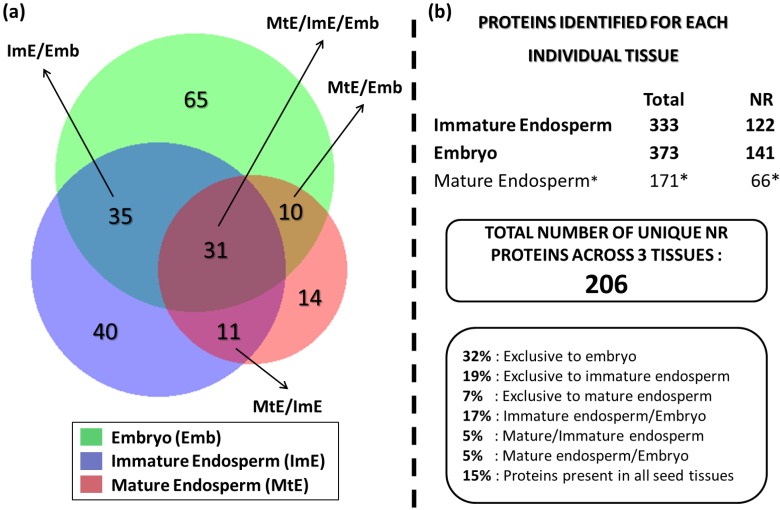
Venn diagram displaying distribution of non-redundant (nr) proteins amongst lotus seed immature endosperm (ImE), mature endosperm (MtE), and embryo (Emb) (**a**); Listing of the total and nr protein matches found for each lotus seed tissue analyzed (**b**); * see reference [17].

As is to be expected, there were many proteins in common found among the immature endosperm and embryo tissues, as well as with the mature endosperm previously analyzed [17]. Amongst all three seed tissues, a total of 206 nr proteins were identified against the plant database (Figure 3). Of these, 31 (15%) were common to all three tissues, 40 (19%) were unique to the immature endosperm, and 65 (32%) were unique to the embryo; only 14 (7%) were exclusively found in the mature endosperm. To note, the larger share of embryo-only proteins is a consequence of the embryo tissue being much more involved in plant metabolism, and therefore is expected to express a larger number of functional proteins than the endosperm, which, especially in its mature phase, has nutrient storage as its primary function. The immature endosperm, as a developing tissue, also expresses a larger number of proteins than its mature form, and also shares a significant number of proteins with the embryo—35 (17%) of the identified ones. Common proteins between mature and immature endosperm only amounted to 5% of the identified ones (same as for between the mature endosperm and embryo). Although, considering that both immature endosperm and immature embryo are much softer and with a higher water content than their mature stages, there is a possibility that some of the proteins in common with the embryo identified in the immature endosperm might have originated from the embryo and diffused through the endosperm, despite the care taken to remove embryo fragments and the endosperm immediately around them in the sample preparation.

### 3.5. Functional Significance of the Identified Seed Proteins

Gene ontology data (biological processes, molecular functions and cellular localization) for all identified proteins were obtained from the UniProtKB database, using the EMBL-EBI (www.ebi.ac.uk) search tool (Table 3).

**Table 3:** List of all 206 non-redundant (nr) proteins found across the three tissues of the lotus seed (embryo, immature endosperm and mature endosperm).

Protein Accession	Protein Description	Tissues ^1^
1433E_TOBAC	14-3-3-like protein E OS = *Nicotiana tabacum*	M/I/E
HSP14_SOYBN	17.5 kDa class I heat shock protein OS = *Glycine max*	I
HSP11_SOLLC	17.8 kDa class I heat shock protein OS = *Solanum lycopersicum*	M
HSP11_PEA	18.1 kDa class I heat shock protein OS = *Pisum sativum*	M/I/E
HSP12_MEDSA	18.2 kDa class I heat shock protein OS = *Medicago sativa*	M/I
HSP11_CHERU	18.3 kDa class I heat shock protein OS = *Chenopodium rubrum*	I
PMG1_ARATH	2,3-bisphosphoglycerate-independent phosphoglycerate mutase 1 OS = *Arabidopsis thaliana*	M/I/E
PRS6A_SOLLC	26S protease regulatory subunit 6A homolog OS = *Solanum lycopersicum*	E
RPN1A_ARATH	26S proteasome non-ATPase regulatory subunit 2 1A OS = *Arabidopsis thaliana*	E
PRS4A_ARATH	26S proteasome regulatory subunit 4 homolog A OS = *Arabidopsis thaliana*	E
BAS1_ORYSJ	2-Cys peroxiredoxin BAS1, chloroplastic OS = *Oryza sativa* subsp. *Japonica*	I
RS102_ARATH	40S ribosomal protein S10-2 OS = *Arabidopsis thaliana*	I
RS13_PEA	40S ribosomal protein S13 OS = *Pisum sativum*	I/E
RS14_CHLRE	40S ribosomal protein S14 OS = *Chlamydomonas reinhardtii*	I/E
RS15A_DAUCA	40S ribosomal protein S15a OS = *Daucus carota*	E
RS16_FRIAG	40S ribosomal protein S16 OS = *Fritillaria agrestis*	I/E
RS18_ARATH	40S ribosomal protein S18 OS = *Arabidopsis thaliana*	I/E
RS193_ARATH	40S ribosomal protein S19-3 OS = *Arabidopsis thaliana*	I/E
RS254_ARATH	40S ribosomal protein S25-4 OS = *Arabidopsis thaliana*	E
RS30_ARATH	40S ribosomal protein S30 OS = *Arabidopsis thaliana*	E
RS33_ARATH	40S ribosomal protein S3-3 OS = *Arabidopsis thaliana*	E
RS3A1_VITVI	40S ribosomal protein S3a-1 OS = *Vitis vinifera*	I
RS4_GOSHI	40S ribosomal protein S4 OS = *Gossypium hirsutum*	I/E
RS5_CICAR	40S ribosomal protein S5 (fragment) OS = *Cicer arietinum*	I/E
RS6_ASPOF	40S ribosomal protein S6 OS = *Asparagus officinalis*	I
RS61_ARATH	40S ribosomal protein S6-1 OS = *Arabidopsis thaliana*	E
RS8_MAIZE	40S ribosomal protein S8 OS = *Zea mays*	E
RS91_ARATH	40S ribosomal protein S9-1 OS = *Arabidopsis thaliana*	M
RS92_ARATH	40S ribosomal protein S9-2 OS = *Arabidopsis thaliana*	E
RSSA_SOYBN	40S ribosomal protein SA OS = *Glycine max*	I/E
METE_ARATH	5-methyltetrahydropteroyltriglutamate--homocysteine methyltransferase OS = *Arabidopsis thaliana*	M/I/E
RLA0_LUPLU	60S acidic ribosomal protein P0 OS = *Lupinus luteus*	I/E
RLA2_PARAR	60S acidic ribosomal protein P2 OS = *Parthenium argentatum*	E
RL10_VITRI	60S ribosomal protein L10 OS = *Vitis riparia*	I
RL11_MEDSA	60S ribosomal protein L11 OS = *Medicago sativa*	E
RL12_PRUAR	60S ribosomal protein L12 OS = *Prunus armeniaca*	I
RL13_TOBAC	60S ribosomal protein L13 OS = *Nicotiana tabacum*	I/E
RL17_MAIZE	60S ribosomal protein L17 OS = *Zea mays*	E
RL182_ARATH	60S ribosomal protein L18-2 OS = *Arabidopsis thaliana*	I
RL18A_CASSA	60S ribosomal protein L18a OS = *Castanea sativa*	E
RL24_PRUAV	60S ribosomal protein L24 OS = *Prunus avium*	E
RL3_ORYSJ	60S ribosomal protein L3 OS = *Oryza sativa* subsp. *Japonica*	I/E
RL4_PRUAR	60S ribosomal protein L4 OS = *Prunus armeniaca*	I/E
RL51_ARATH	60S ribosomal protein L5-1 OS = *Arabidopsis thaliana*	I/E
RL6_MESCR	60S ribosomal protein L6 OS = *Mesembryanthemum crystallinum*	I
RL7A1_ARATH	60S ribosomal protein L7a-1 OS = *Arabidopsis thaliana*	E
ACOC_CUCMA	Aconitate hydratase, cytoplasmic OS = *Cucurbita maxima*	M/E
ACT_GOSHI	Actin OS = *Gossypium hirsutum*	I/E
ACT1_ORYSI	Actin-1 OS = *Oryza sativa* subsp. *Indica*	M
ACT12_SOLTU	Actin-100 (fragment) OS = *Solanum tuberosum*	M/E
ACT1_SOLLC	Actin-41 (fragment) OS = *Solanum lycopersicum*	M
ACT7_ARATH	Actin-7 OS = *Arabidopsis thaliana*	M
SAHH_MEDSA	Adenosylhomocysteinase OS = *Medicago sativa*	M/I/E
ADT1_GOSHI	ADP, ATP carrier protein 1, mitochondrial OS = *Gossypium hirsutum*	M/I/E
ARF_VIGUN	ADP-ribosylation factor OS = *Vigna unguiculata*	E
SYA_ARATH	Alanine--tRNA ligase OS = *Arabidopsis thaliana*	E
ADH1_SOLTU	Alcohol dehydrogenase 1 OS = *Solanum tuberosum*	M/I
PHSL_IPOBA	Alpha-1,4 glucan phosphorylase L isozyme, chloroplastic/amyloplastic OS = *Ipomoea batatas*	M/E
PHSH_ARATH	Alpha-glucan phosphorylase, H isozyme OS = *Arabidopsis thaliana*	M/I
GCST_PEA	Aminomethyltransferase, mitochondrial OS = *Pisum sativum*	I/E
ANXD1_ARATH	Annexin D1 OS = *Arabidopsis thaliana*	M/I/E
ANX4_FRAAN	Annexin-like protein RJ4 OS = *Fragaria ananassa*	E
CYF_AETCO	Apocytochrome f OS = *Aethionema cordifolium*	I
AATM_LUPAN	Aspartate aminotransferase P2, mitochondrial (fragment) OS = *Lupinus angustifolius*	E
AATM_LUPAN	Aspartate aminotransferase P2, mitochondrial (fragment) OS = *Lupinus angustifolius*	I
AAT3_ARATH	Aspartate aminotransferase, chloroplastic OS = *Arabidopsis thaliana*	I/E
AATC_DAUCA	Aspartate aminotransferase, cytoplasmic OS = *Daucus carota*	E
PYRB_ARATH	Aspartate carbamoyltransferase, chloroplastic OS = *Arabidopsis thaliana*	I
ATPAM_HELAN	ATP synthase subunit alpha, mitochondrial OS = *Helianthus annuus*	M/I/E
ATPBM_NICPL	ATP synthase subunit beta, mitochondrial OS = *Nicotiana plumbaginifolia*	M/I/E
ACLB1_ORYSJ	ATP-citrate synthase beta chain protein 1 OS = *Oryza sativa* subsp. *Japonica*	E
CLPA_BRANA	ATP-dependent Clp protease ATP-binding subunit clpA homolog, chloroplastic (fragment) OS = *Brassica napus*	I
APBLB_ARATH	Beta-adaptin-like protein B OS = *Arabidopsis thaliana*	E
ENO2_ARATH	Bifunctional enolase 2/transcriptional activator OS = *Arabidopsis thaliana*	I/E
ACCC2_POPTR	Biotin carboxylase 2, chloroplastic OS = *Populus trichocarpa*	E
CALSB_ARATH	Callose synthase 11 OS = *Arabidopsis thaliana*	E
CALX2_ARATH	Calnexin homolog 2 OS = *Arabidopsis thaliana*	E
CALR_BERST	Calreticulin OS = *Berberis stolonifera*	I
CATA2_RICCO	Catalase isozyme 2 OS = *Ricinus communis*	E
CD48A_ARATH	Cell division control protein 48 homolog A OS = *Arabidopsis thaliana*	M/I/E
CLPB1_ARATH	Chaperone protein ClpB1 OS = *Arabidopsis thaliana*	E
CLPC1_ARATH	Chaperone protein ClpC1, chloroplastic OS = *Arabidopsis thaliana*	I
CPNA1_ARATH	Chaperonin 60 subunit alpha 1, chloroplastic OS = *Arabidopsis thaliana*	I/E
CPNB3_ARATH	Chaperonin 60 subunit beta 3, chloroplastic OS = *Arabidopsis thaliana*	E
CH60A_ARATH	Chaperonin CPN60, mitochondrial OS = *Arabidopsis thaliana*	M/I/E
CB2_PHYPA	Chlorophyll a-b binding protein, chloroplastic OS = *Physcomitrella patens* subsp. *patens*	I
HSP7E_SPIOL	Chloroplast envelope membrane 70 kDa heat shock-related protein OS = *Spinacia oleracea*	M/I/E
HSP12_SOYBN	Class I heat shock protein (fragment) OS = *Glycine max*	I
CLAH1_ARATH	Clathrin heavy chain 1 OS = *Arabidopsis thaliana*	I/E
COPA1_ARATH	Coatomer subunit alpha-1 OS = *Arabidopsis thaliana*	E
COB21_ORYSJ	Coatomer subunit beta-1 OS = *Oryza sativa* subsp. *Japonica*	I
RH2_ORYSJ	DEAD-box ATP-dependent RNA helicase 2 OS = *Oryza sativa* subsp. *Japonica*	M
DIM_PEA	Delta(24)-sterol reductase OS = *Pisum sativum*	E
DLDH2_ARATH	Dihydrolipoyl dehydrogenase 2, mitochondrial OS = *Arabidopsis thaliana*	I
OPD22_ARATH	Dihydrolipoyllysine-residue acetyltransferase component 2 of pyruvate dehydrogenase complex, mitochondrial OS = *Arabidopsis thaliana*	E
EF1A_TOBAC	Elongation factor 1-alpha OS = *Nicotiana tabacum*	M/I/E
EF1D1_ORYSJ	Elongation factor 1-delta 1 OS = *Oryza sativa* subsp*.* *Japonica*	E
EF1G2_ORYSJ	Elongation factor 1-gamma 2 OS = *Oryza sativa* subsp. *Japonica*	M/I/E
EF2_BETVU	Elongation factor 2 OS = *Beta vulgaris*	M/I/E
EFTM_ARATH	Elongation factor Tu, mitochondrial OS = *Arabidopsis thaliana*	E
ENPL_CATRO	Endoplasmin homolog OS = *Catharanthus roseus*	M/I/E
ENO1_HEVBR	Enolase 1 OS = *Hevea brasiliensis*	M/I/E
IF4A1_ARATH	Eukaryotic initiation factor 4A-1 OS = *Arabidopsis thaliana*	M/I/E
ERF1X_ARATH	Eukaryotic peptide chain release factor subunit 1-1 OS = *Arabidopsis thaliana*	E
IF5A1_ARATH	Eukaryotic translation initiation factor 5A-1 OS = *Arabidopsis thaliana*	E
ALF_CICAR	Fructose-bisphosphate aldolase, cytoplasmic isozyme OS = *Cicer arietinum*	M/I/E
RFS_ORYSJ	Galactinol--sucrose galactosyltransferase OS = *Oryza sativa* subsp. *Japonica*	I
GME2_ORYSJ	GDP-mannose 3,5-epimerase 2 OS = *Oryza sativa* subsp. *Japonica*	E
GMD1_ARATH	GDP-mannose 4,6 dehydratase 1 OS = *Arabidopsis thaliana*	E
GRDH1_ARATH	Glucose and ribitol dehydrogenase homolog 1 OS = *Arabidopsis thaliana*	M/I
GLGS_BETVU	Glucose-1-phosphate adenylyltransferase small subunit, chloroplastic/amyloplastic (fragment) OS = *Beta vulgaris*	M
G6PI2_CLACO	Glucose-6-phosphate isomerase, cytosolic 2 OS = *Clarkia concinna*	M/E
GPT2_ARATH	Glucose-6-phosphate/phosphate translocator 2, chloroplastic OS = *Arabidopsis thaliana*	M
GLN11_ORYSJ	Glutamine synthetase cytosolic isozyme 1-1 OS = *Oryza sativa* subsp. *Japonica*	E
G3PC_ANTMA	Glyceraldehyde-3-phosphate dehydrogenase, cytosolic OS = *Antirrhinum majus*	M/I/E
SYGM1_ARATH	Glycine--tRNA ligase 1, mitochondrial OS = *Arabidopsis thaliana*	E
SSG1_HORVU	Granule-bound starch synthase 1, chloroplastic/amyloplastic OS = *Hordeum vulgare*	M/I
RAN_VICFA	GTP-binding nuclear protein Ran/TC4 OS = *Vicia faba*	M/I/E
RAN3_ORYSI	GTP-binding nuclear protein Ran-3 OS = *Oryza sativa* subsp. *Indica*	E
YPTC1_CHLRE	GTP-binding protein YPTC1 OS = *Chlamydomonas reinhardtii*	E
GBLPA_ORYSJ	Guanine nucleotide-binding protein subunit beta-like protein A OS = *Oryza sativa* subsp. Japonica	I
HSP7L_ARATH	Heat shock 70 kDa protein 12 OS = *Arabidopsis thaliana*	I/E
HSP7O_ARATH	Heat shock 70 kDa protein 14 OS = *Arabidopsis thaliana*	I/E
HSP7N_ARATH	Heat shock 70 kDa protein 18 OS = *Arabidopsis thaliana*	I
HSP7D_ARATH	Heat shock 70 kDa protein 4 OS = *Arabidopsis thaliana*	I
HSP7F_ARATH	Heat shock 70 kDa protein 6, chloroplastic OS = *Arabidopsis thaliana*	I
HSP7G_ARATH	Heat shock 70 kDa protein 7, chloroplastic OS = *Arabidopsis thaliana*	E
HSP70_DAUCA	Heat shock 70 kDa protein OS = *Daucus carota*	M/I/E
HSP7M_PHAVU	Heat shock 70 kDa protein, mitochondrial OS = *Phaseolus vulgaris*	I/E
HSP80_SOLLC	Heat shock cognate protein 80 OS = *Solanum lycopersicum*	M/I
HS101_ARATH	Heat shock protein 101 OS = *Arabidopsis thaliana*	M
HS101_ORYSJ	Heat shock protein 101 OS = *Oryza sativa* subsp. *Japonica*	M
HSP81_ORYSI	Heat shock protein 81-1 OS = *Oryza sativa* subsp. *Indica*	M/I/E
HSP82_TOBAC	Heat shock protein 82 (fragment) OS = *Nicotiana tabacum*	M
HSP82_MAIZE	Heat shock protein 82 OS = *Zea mays*	M/I/E
HSP83_IPONI	Heat shock protein 83 OS = *Ipomoea nil*	M/I/E
HS901_ARATH	Heat shock protein 90-1 OS = *Arabidopsis thaliana*	E
HS903_ARATH	Heat shock protein 90-3 OS = *Arabidopsis thaliana*	I
H2AX_CICAR	Histone H2AX OS = *Cicer arietinum*	I
H2B_GOSHI	Histone H2B OS = *Gossypium hirsutum*	I/E
H4_ARATH	Histone H4 OS = *Arabidopsis thaliana*	I/E
IDHC_TOBAC	Isocitrate dehydrogenase [NADP] OS = *Nicotiana tabacum*	E
ILV5_ARATH	Ketol-acid reductoisomerase, chloroplastic OS = *Arabidopsis thaliana*	E
APX1_ORYSJ	L-ascorbate peroxidase 1, cytosolic OS = *Oryza sativa* subsp. *Japonica*	E
LE194_HORVU	Late embryogenesis abundant protein B19.4 OS = *Hordeum vulgare*	I
AMPL1_ARATH	Leucine aminopeptidase 1 OS = *Arabidopsis thaliana*	M/I
BIP4_TOBAC	Luminal-binding protein OS = *Nicotiana tabacum*	M/I/E
MDHC2_ARATH	Malate dehydrogenase, cytoplasmic 2 OS = *Arabidopsis thaliana*	M/E
MDHM_CITLA	Malate dehydrogenase, mitochondrial OS = *Citrullus lanatus*	M/I/E
MPPA_SOLTU	Mitochondrial-processing peptidase subunit alpha OS = *Solanum tuberosum*	E
MDAR_SOLLC	Monodehydroascorbate reductase OS = *Solanum lycopersicum*	I/E
MAOX_POPTR	NADP-dependent malic enzyme OS = *Populus trichocarpa*	M
NDK1_ARATH	Nucleoside diphosphate kinase 1 OS = *Arabidopsis thaliana*	M/I/E
FKB62_ARATH	Peptidyl-prolyl cis-trans isomerase FKBP62 OS = *Arabidopsis thaliana*	I/E
PER1B_ARMRU	Peroxidase C1B OS = *Armoracia rusticana*	I
CAPPC_FLATR	Phosphoenolpyruvate carboxylase 2 OS = *Flaveria trinervia*	E
PGMC_PEA	Phosphoglucomutase, cytoplasmic OS = *Pisum sativum*	M/I/E
PGKH_TOBAC	Phosphoglycerate kinase, chloroplastic OS = *Nicotiana tabacum*	M/I
PGKY_TOBAC	Phosphoglycerate kinase, cytosolic OS = *Nicotiana tabacum*	M/E
SERC_SPIOL	Phosphoserine aminotransferase, chloroplastic OS = *Spinacia oleracea*	E
PDR4_ORYSJ	Pleiotropic drug resistance protein 4 OS = *Oryza sativa s*ubsp. *Japonica*	E
PARP3_SOYBN	Poly [ADP-ribose] polymerase 3 OS = *Glycine max*	I
UBIQP_ACECL	Polyubiquitin (fragment) OS = *Acetabularia cliftonii*	I
UBQ12_ARATH	Polyubiquitin 12 OS = *Arabidopsis thaliana*	E
PMG2_ARATH	Probable 2,3-bisphosphoglycerate-independent phosphoglycerate mutase 2 OS = *Arabidopsis thaliana*	M/I
SSG1_ARATH	Probable granule-bound starch synthase 1, chloroplastic/amyloplastic OS = *Arabidopsis thaliana*	I
H2B1_MEDTR	Probable histone H2B.1 OS = *Medicago truncatula*	I
PDIA6_MEDSA	Probable protein disulfide-isomerase A6 OS = *Medicago sativa*	I
Y1497_ARATH	Probable receptor-like protein kinase At1g49730 OS = *Arabidopsis thaliana*	I
PROF3_ARATH	Profilin-3 OS = *Arabidopsis thaliana*	E
PSA3_ARATH	Proteasome subunit alpha type-3 OS = *Arabidopsis thaliana*	E
PDI21_ORYSJ	Protein disulfide isomerase-like 2-1 OS = *Oryza sativa* subsp. *Japonica*	I
PDI21_ARATH	Protein disulfide-isomerase like 2-1 OS = *Arabidopsis thaliana*	M/I/E
ACT5_ARATH	Putative actin-5 OS = *Arabidopsis thaliana*	I/E
YCF1_IPOPU	Putative membrane protein ycf1 OS = *Ipomoea purpurea*	M/I
AVP_VIGRR	Pyrophosphate-energized vacuolar membrane proton pump OS = *Vigna radiata* var. *radiata*	I/E
PDC1_TOBAC	Pyruvate decarboxylase isozyme 1 (fragment) OS = *Nicotiana tabacum*	M/I
KPYC_SOYBN	Pyruvate kinase, cytosolic isozyme OS = *Glycine max*	M/I/E
PPDK2_ORYSJ	Pyruvate, phosphate dikinase 2 OS = *Oryza sativa* subsp. *Japonica*	M
PPDK_FLABR	Pyruvate, phosphate dikinase, chloroplastic OS = *Flaveria brownii*	M/E
RAA1D_ARATH	Ras-related protein RABA1d OS = *Arabidopsis thaliana*	E
RBL_MAIZE	Ribulose bisphosphate carboxylase large chain OS = *Zea mays*	I
RUBA_RICCO	RuBisCO large subunit-binding protein subunit alpha (fragment) OS = *Ricinus communis*	I/E
RUBB_PEA	RuBisCO large subunit-binding protein subunit beta, chloroplastic OS = *Pisum sativum*	E
METK4_POPTR	S-adenosylmethionine synthase 4 OS = *Populus trichocarpa*	E
SAPK6_ORYSJ	Serine/threonine-protein kinase SAPK6 OS = *Oryza sativa* subsp*. Japonica*	E
HSP7S_SPIOL	Stromal 70 kDa heat shock-related protein, chloroplastic (fragment) OS = *Spinacia oleracea*	I/E
SUSY_SOYBN	Sucrose synthase OS = *Glycine max*	I/E
SODM_HEVBR	Superoxide dismutase [Mn], mitochondrial OS = *Hevea brasiliensis*	E
TCPA_ARATH	T-complex protein 1 subunit alpha OS = *Arabidopsis thaliana*	I/E
TCPE_ARATH	T-complex protein 1 subunit epsilon OS = *Arabidopsis thaliana*	M/E
TKTC_SPIOL	Transketolase, chloroplastic OS = *Spinacia oleracea*	E
TCTP_TOBAC	Translationally-controlled tumor protein homolog OS = *Nicotiana tabacum*	M
TPIS_MAIZE	Triosephosphate isomerase, cytosolic OS = *Zea mays*	I/E
TBA_PRUDU	Tubulin alpha chain OS = *Prunus dulcis*	I
TBB_HORVU	Tubulin beta chain OS = *Hordeum vulgare*	E
UBIQ_ARATH	Ubiquitin OS = *Arabidopsis thaliana*	M/E
RL40A_ARATH	Ubiquitin-60S ribosomal protein L40-1 OS = *Arabidopsis thaliana*	I/E
RGP1_ORYSJ	UDP-arabinopyranose mutase 1 OS = *Oryza sativa* subsp. *Japonica*	E
UGDH_SOYBN	UDP-glucose 6-dehydrogenase OS = *Glycine max*	E
UREA_CANEN	Urease OS = *Canavalia ensiformis*	I
UGPA1_ARATH	UTP--glucose-1-phosphate uridylyltransferase 1 OS = *Arabidopsis thaliana*	M/E
VATA_GOSHI	V-type proton ATPase catalytic subunit A OS = *Gossypium hirsutum*	I/E
VATB2_GOSHI	V-type proton ATPase subunit B 2 (fragment) OS = *Gossypium hirsutum*	E
VATB1_ARATH	V-type proton ATPase subunit B1 OS = *Arabidopsis thaliana*	E
WIT2_ARATH	WPP domain-interacting tail-anchored protein 2 OS = *Arabidopsis thaliana*	I

^1^ M: Mature endosperm; I: Immature endosperm; E: Embryo

**Figure 4:**
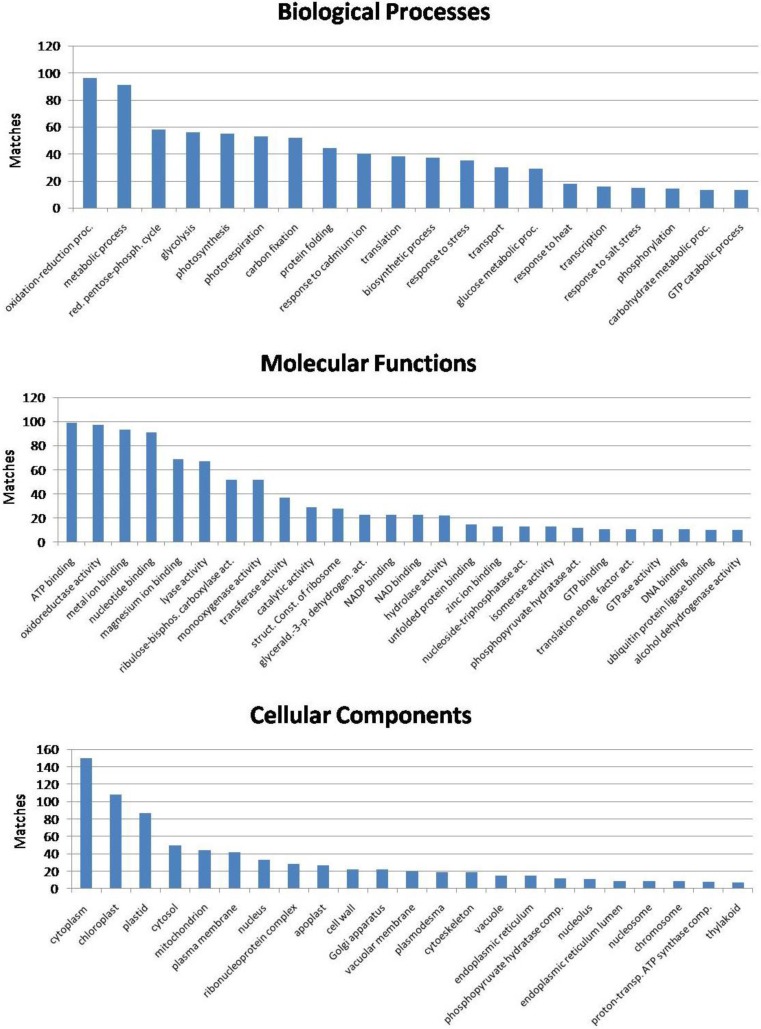
Distribution of the top gene ontology (GO) data for lotus immature endosperm proteome based on 1-DGE-MS analysis.

Analysis of the annotations referent to the immature endosperm revealed that functions related to protein synthesis (translation, protein folding and polymerization, *etc*.), general metabolism (amino acid, carbon fixation) and carbohydrate metabolism (glycolysis, etc.) are all considerably represented, with the proteins in the first category being relatively more numerous (Figure 4).

**Figure 5:**
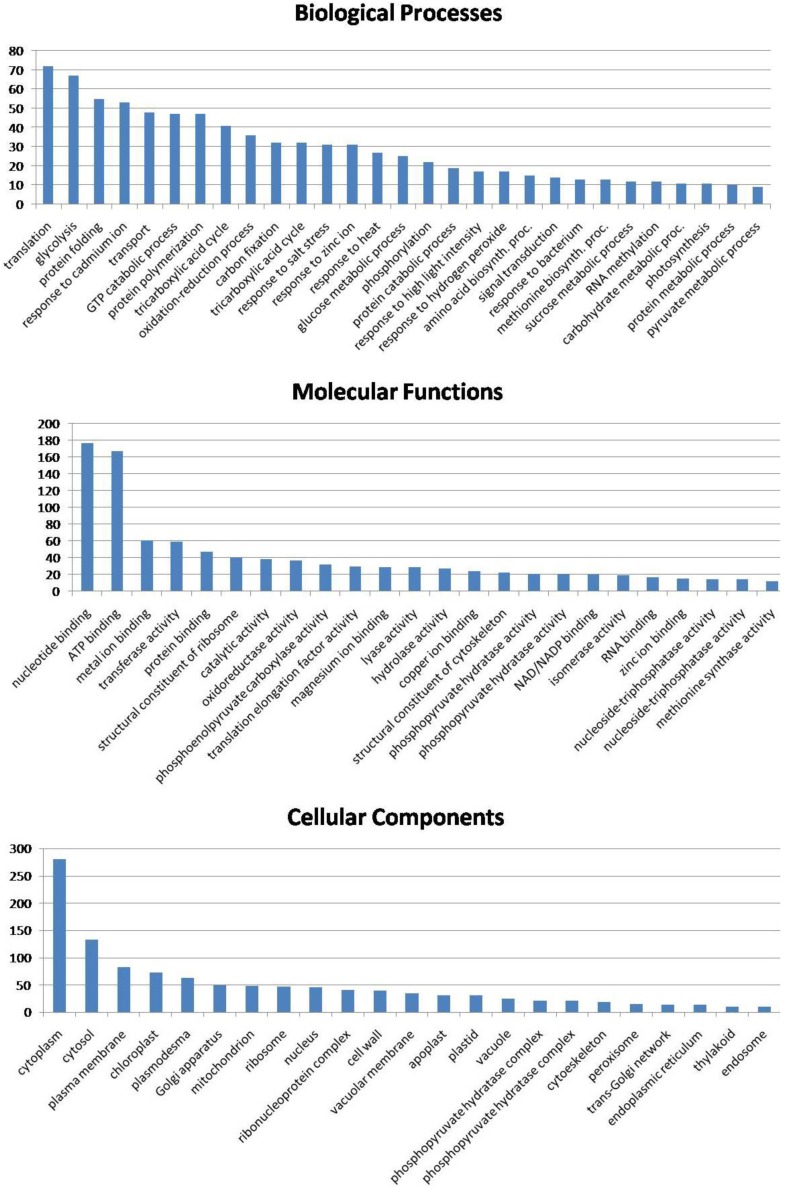
Distribution of the top gene ontology (GO) data for lotus embryo proteome based on 1-DGE-MS analysis.

On the other hand, the embryo proteome shows considerable prevalence of proteins involved in protein synthesis, followed then by carbohydrate and general metabolism processes (Figure 5).

### 3.6. Biological Function of the Identified Seed Proteins

**Figure 6:**
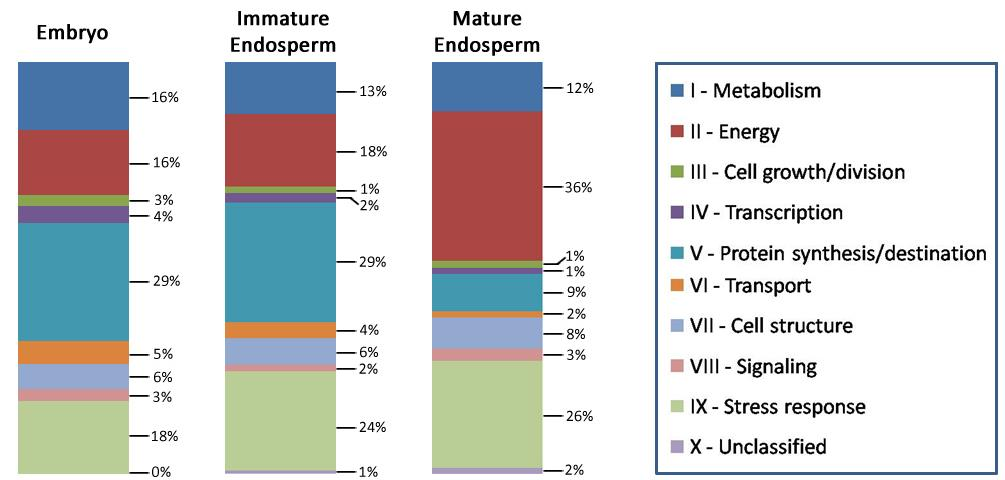
Bar charts displaying the division according to functional categories, of the non-redundant (nr) protein matches found in the lotus seed embryo, immature endosperm, and mature endosperm, as determined by 1-DGE-MS.

Furthermore, the nr protein matches were also classified according to their broader biological function [22,23], divided into 10 categories: metabolism, energy, cell growth/division, transcription, protein synthesis/destination, transporters, cell structure, signal transduction, stress response, and unclassified (Figure 6).

A comparison of the distribution of protein functionality between the seed immature endosperm and embryo, and the previous results obtained from the mature endosperm shows that immature endosperm and embryo have a quite similar functionality profile of the mature endosperm. However, in the embryo the identified proteins related to general cell housekeeping functions (non-energy metabolism, cell growth, transcription, transport, and signaling) were slightly more apparent than in the immature endosperm. In contrast with the mature endosperm, both immature endosperm and embryo show a larger percentage of the identified proteins related to protein synthesis. This correlated well with the fact that the tissues are either in a growing phase, *i.e.*, immature endosperm or have growth as their main function, *i.e.*, embryo. The mature endosperm, on the other hand, having its primary function as energy and nutrient storage, has the larger share of its proteins related to energy metabolism. A common element for all the lotus seed tissues is the large presence of stress-/defense-related proteins across all samples.

### 3.7. Lotus Seed Proteome Compared with Other Seed Proteomes

Unlike some seeds, such as tomato, where non-germinating embryo and endosperm were shown to have very similar proteomes [24], the analysis of lotus seed proteomes showed some remarkable difference in proteins identified/function between the non-germinating embryo and mature endosperm. Contrary to other seed proteomes like *Jatropha curcas* [23] and sugarbeet [25], the lotus embryo in its pre-germination stage did not seem to have a considerably higher expression of metabolism- and energy-related proteins compared to the mature endosperm. Structural proteins, however, did seem to be at least slightly more represented in the endosperm, as in the case of *J. curcas*. Compared with other embryo proteomes, such as *Brassica campestri* [26], and sugarbeet, the lotus embryo appears to have a larger percentage of proteins related to protein synthesis in comparison to primary and energy metabolism, as well as a much greater presence of defense related proteins. We further discuss below the key proteins identified in this study.

### 3.8. Key Proteins of the Lotus Immature Seed Endosperm

**Figure 7:**
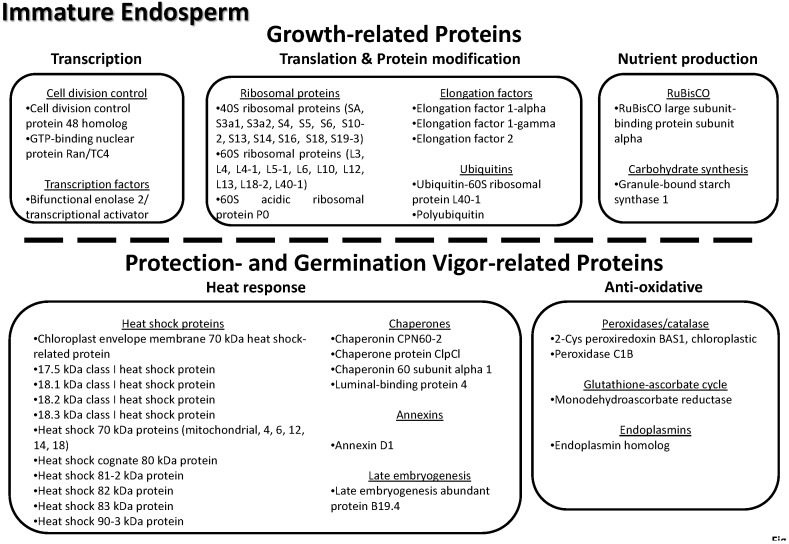
Key functional proteins identified in the lotus seed immature endosperm, and subdivided according to their role in plant metabolism.

Contrary to the mature endosperm, the key functional proteins identified in the lotus immature endosperm mostly consisted of proteins related to plant growth and development (Figure 7).

Amongst the identified proteins were several transcription proteins (cell division control and transcription factors), translation (ribosomal) proteins, post-translational modification proteins (elongation factors and ubiquitins) and nutrient production proteins (RuBisCO subunits and sucrose synthase). Many stress response- and plant defense-related proteins were also present in the immature endosperm. Of these, the largest subgroup is the heat shock response proteins (high- and low-molecular weight heat shock proteins (HSPs), as well as chaperone and annexin proteins). Anti-oxidative stress (peroxidases, endoplasmin, and monodehydroascorbate reductase) are also present, more so than in the mature endosperm (see below section). Proteins related to carbohydrate metabolism are also present in the immature endosperm, but in a smaller number.

### 3.9. Key Proteins Previously Identified in the Lotus Mature Seed Endosperm

**Figure 8:**
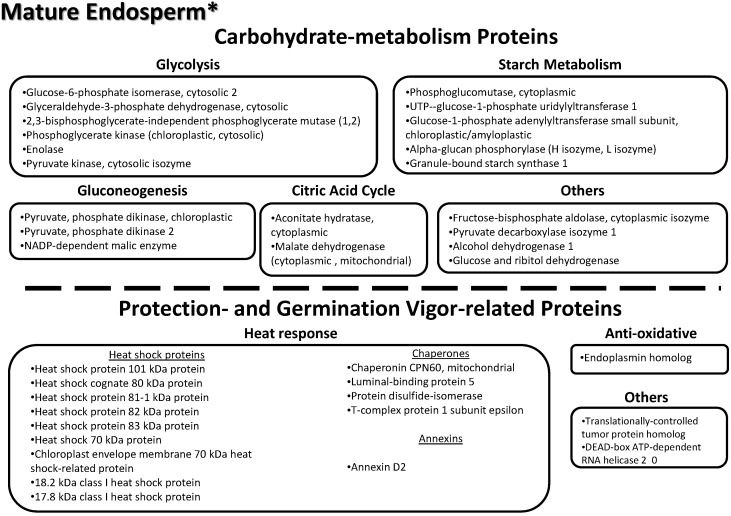
Key functional proteins identified in the lotus seed mature endosperm, and subdivided according to their role in plant metabolism. * for original protein lists, see reference [17].

In the case of mature endosperm proteome [17], the two most significant groups of proteins identified were related to energy/carbohydrate metabolism, and stress response and plant protection (Figure 8). In the first group, several proteins that are part of glycolysis, gluconeogenesis, citric acid cycle and starch metabolism including other carbohydrate metabolism proteins, were identified. Of the stress response proteins, HSPs, along with other heat response proteins (chaperones, annexin), constituted the most numerous category. Anti-oxidative stress proteins were not greatly represented. Of note is the identification of storage proteins (such as globulins, castanins) for the mature endosperm by 2-D MS and N-terminal sequencing, but not by 1-D MS [17,27], which might indicate a possible detection gap of this technique.

### 3.10. Proteome Changes between Mature and Immature Stages of the Endosperm

**Figure 9:**
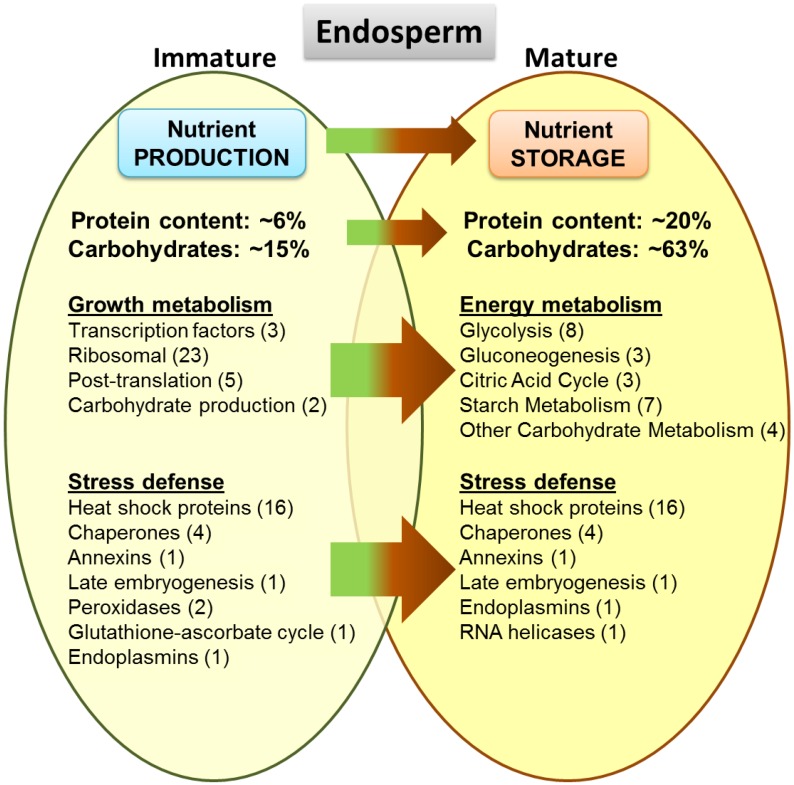
Depiction of the changes in biological function, nutrient content and functional proteome composition from the immature (left) to the mature (right) endosperm in the lotus seed.

Despite constituting the endosperm tissue samples, protein extracts from the mature and immature seed presented a notably different proteome composition (Figure 9).

This reflects the changes the endosperm undergoes during the maturation process, where it develops from a soft wet tissue to a dry one with a large amount per weight of both carbohydrates and proteins [6]. The endosperm’s main role in the seed is as a nutrient storage tissue, so it is expected that during the maturation phase, these nutrients are going to be produced for later storage, hence the larger number of functional proteins related to the protein and carbohydrate synthesis categories. In the mature endosperm, a large percentage of the total protein content is expected to be seed storage proteins (SSPs). Although not many SSPs were identified by MS analysis of the mature endosperm, several possible matches were found by N-terminal sequencing analysis [27]. The prevalence of carbohydrate metabolism proteins amongst the identified functional proteins in the mature endosperm could be a result of production in the late maturation stage, with such proteins playing a quasi-dormant role in managing the nutrient content of the seed before and during germination.

### 3.11. Key Proteins of the Lotus Seed Embryo

**Figure 10:**
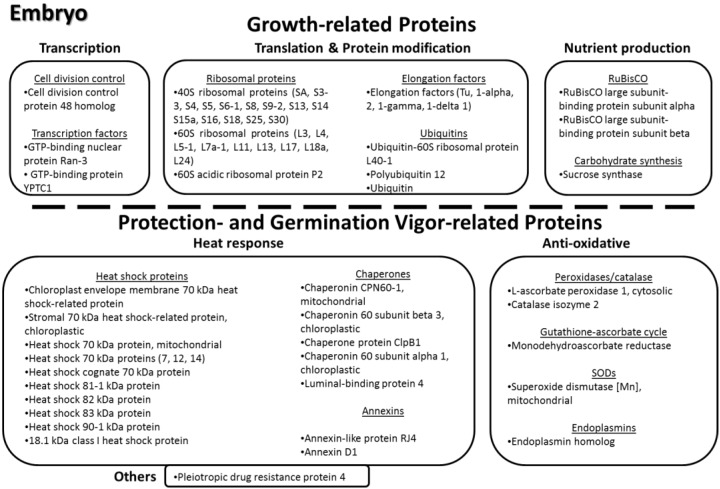
Key functional proteins identified in the lotus seed embryo, and subdivided according to their role in plant metabolism.

In the case of the embryo proteins identified by database matching, the distribution of key proteins was similar to that of the immature endosperm, in that they can be divided in the same main groups: proteins related to plant growth, and proteins responsible for plant protection and germination vigor (Figure 10). Of the first group, those also include the same subgroups of transcription, translation, and post-translation proteins as well as nutrient production proteins. In the case of stress/defense-related proteins, the embryo was also found to possess the largest number heat shock response proteins (12 HSPs, mostly of high-molecular weight, five chaperone proteins and two annexins). However, the embryo also contained a larger number of anti-oxidative stress proteins, including l-ascorbate peroxidase, catalase, monodehydroascorbate reductase, superoxide dismutase [Mn], and endoplasmin. *S*-adenosylmethionine synthase and adenosylhomocysteinase (also found in the endosperm tissues), and two proteins from the active methyl cycle, which is of great importance to plant metabolism as well as their nutritional value [28], were also identified in the lotus embryo.

## 4. Conclusions

Analysis of protein extracts from the lotus seed embryo and immature seed endosperm was performed following 1-DGE separation in conjunction with LC-MS/MS analysis. This “bottom-up” proteomics analysis, represented by the SDS-PAGE technique, has been shown to be a good approach for identifying the lotus seed proteins [17]. For both tissues, a great number of proteins were identified by database matching. A total of 141 nr protein matches were identified in the embryo, and 122 in the immature endosperm. Together with the 66 proteins previously identified for the mature endosperm, a total of 206 nr proteins have been identified to date.

Combined datasets are a resource in itself towards complete proteomics analysis of lotus seeds and plants. By producing more extensive datasets, these results help toward forming a complete proteomic picture of the lotus seeds. The analysis of protein makeup and functionality across different tissues within the seed also permits a comparison of metabolic functions across different tissues and developmental stages of the lotus seed, as well as allowing for the comparison with similar tissues from other plants. Furthermore, the identification of proteins of interest—such as key proteins in the metabolism, proteins that confer resistance against stress or germination vigor—opens up several possibilities for more specific studies on these proteins and their possible use in producing transgenic varieties of interest.

Future work will both strive to expand the lotus proteome to other developmentally important tissues, such as seedling and rhizome, as well as to isolate and characterize functional proteins of interest in the seed proteome. Moreover, 2-DGE-MS analysis of individual proteins, especially by *de novo* proteome analysis techniques, coupled with genome comparison, can help obtain more detailed sequences of lotus-specific proteins, since the high taxonomical distance of the lotus in relation to other modern plants hinders the achievement of higher homology values when database-matching proteins.
